# An extensive dataset on micromechanical behavior and microstructure of 1000 days old B/S-based alkali-activated material

**DOI:** 10.1016/j.dib.2023.109131

**Published:** 2023-04-07

**Authors:** Paulo Roberto Ribeiro Soares Junior, Ederson Jose da Silva, Augusto Cesar da Silva Bezerra

**Affiliations:** a Department of Civil Engineering, Federal Center for Technological Education of Minas Gerais, Av. Amazonas, 5253, Belo Horizonte/MG, 30.421-169, Brazil; b Department of Materials Engineering, Federal Center for Technological Education of Minas Gerais, Av. Amazonas, 5253, Belo Horizonte/MG, 30.421-169, Brazil

**Keywords:** Alkali-activated material, Biomass ash, Silica fume, Micromechanical behavior, Microstructure

## Abstract

This dataset contains extensive results on micromechanical behavior and microstructure of alkali-activated materials (AAM) with biomass ash (B) and silica fume (S) precursors. The data were collected at the laboratories of the Federal Center for Technological Education of Minas Gerais (CEFET-MG) in Brazil. Scanning electron microscope (SEM), optical microscopy (OM), and nanoindentation with instrumented penetration (NI) were performed from AAM in the hardened state and advanced age (1000 days). Data include loading curves, hardness, module of elasticity, and microstructure. Data may be useful for researchers and engineers in designing new alternative binders with improved durability.

**Specifications Table**

**Table:** 

Subject	Civil and Structural Engineering
Specific subject area	Sustainable construction materials
Type of data	Table
	Image
	Figure
How the data were acquired	The data were acquired in the materials laboratory using the following instruments: - Scanning electron microscope (SEM), Hitachi, TM3000, detection of backscattered electrons, magnification of x15 to x30.000, and accelerating of 5 and 15kV. - Optical microscope (OM) binocular, Kontrol, with polarized light and camera model MCDE-5 A. - Ultra-microdurometer (UMH), Shimadzu, DUH-211S, pyramid indenter (Vickers) and microscope optical system (x500), objective (x50) and ocular (x10).
Data format	Raw Analyzed
Description of data collection	After 1000 days of curing, specimens fragments were embedded in resin, sanded, polished, and tested by SEM, OM, and UMH. The data were collected under laboratory conditions (humidity and temperature approximately 60% of 25°C), and the main micromechanical properties measured (hardness and modulus).
Data source location	Data were collected at the Federal Center for Technological Education of Minas Gerais, Belo Horizonte, Minas Gerais, Brazil. (19°55′49″S; 43°58′43″W)
Data accessibility	With this article and an online dataset: Repository name: Mendeley Data Title: *Raw and analyzed data on the micromechanical behavior and microstructure of 1000-day-old alkali-activated materials* DOI: https://doi.org/10.17632/fr6cmtnj2w Direct URL: https://data.mendeley.com/datasets/fr6cmtnj2w Note: Please note that the final version of the dataset is *version 3*, which was published on 23 Mar 23 and is widely available in the repository.

**Value of the Data**

• This dataset can help researchers in the development of alternative cementitious materials (clinker-free), given the use of waste-based raw materials.
• These data can be useful for engineers in designing more durable structures, considering the collection of data on 1000-day samples.
• The data help elucidate the micromechanical behavior and microstructure of AAM at advanced ages using SEM, OM, and UMH techniques.
• The contribution of unpublished data can help expand the AAM knowledge frontier.

## 1 Objective

The alkali-activated materials (AAM) are alternative binders usually produced from waste. This aspect encourages sustainability issues, promoting the recovery of waste that would otherwise be disposed of in the environment [1]. Over the last few years, much has been studied about AAM with exceptional findings. However, durability in different environments needs to be better studied [2]. In this scenario, the present work aims to consolidate a vast amount of data on waste-based AAM with advanced ages of 1000 days.

This dataset was organized into two main sections on (i) micromechanical behavior and (ii) microstructure. Various dosages were used, as described in the Methodology (Section 2). The raw data were deposited online in the Mendeley Data and are available to readers [3].

## 2 Data Description

### 2.1 Micromechanical behavior

#### 2.1.1 AAM 60B40S 0 mol/L (sample T1)

**Fig. 1:**
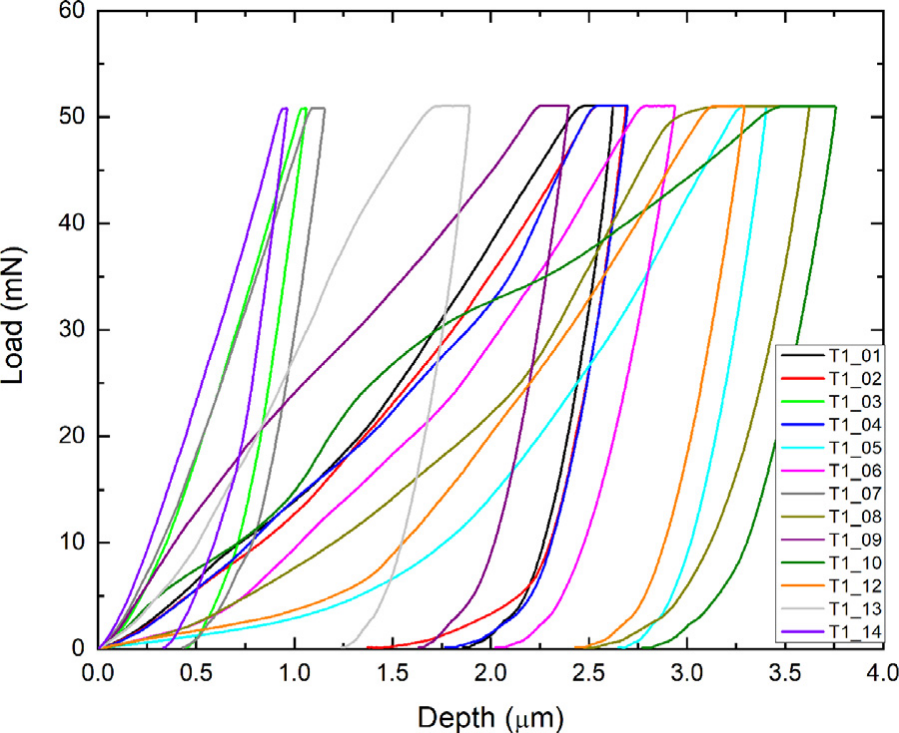
P-h curves for sample T1.

The Fig. 1 shows the load-depth (P-h) curves for sample T1 (60B40S 0 mol/L), grouped in a single graph.

**Fig. 2:**
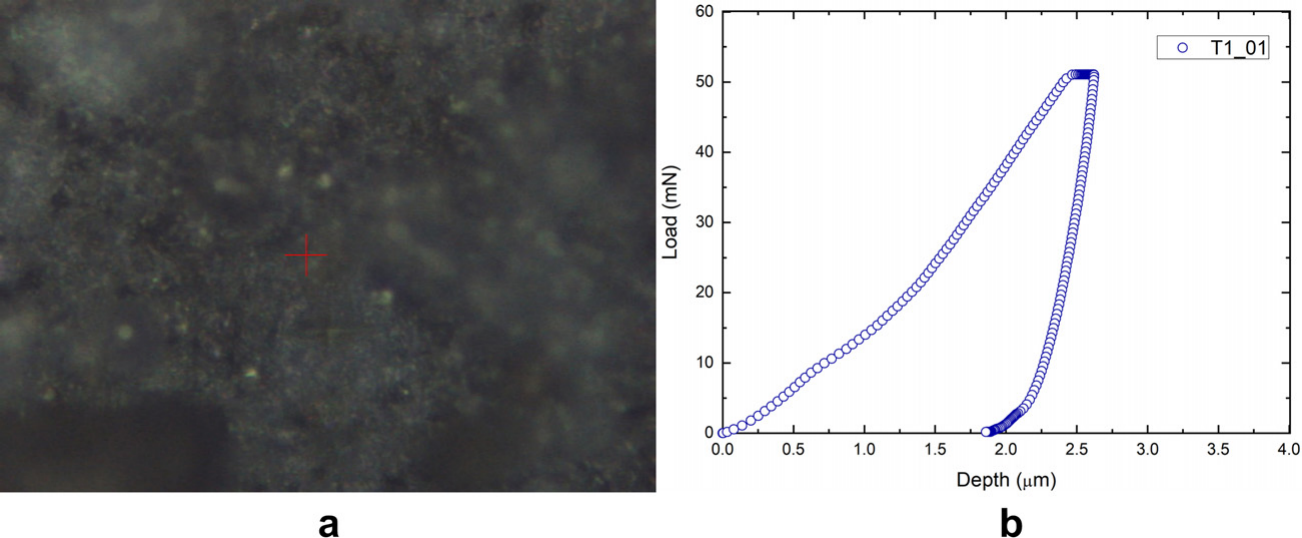
Nanoindentation test results of AAM 60B40S 0 mol/L (sample T1), surface T1_01 (a) OM image and (b) P-h curve.

The Fig. 2 shows the nanoindentation test results of AAM 60B40S 0 mol/L (sample T1) for surface T1_01. The Fig. 2(a) shows OM image and Fig. 2(b) shows P-h curve. The Table 1 shows micromechanical properties.

**Table 1:** Micromechanical properties of AAM 60B40S 0 mol/L (sampleT1), surface T1_01.

Micromechanical properties	
Hardness (Hv)	94,68
Modulus of elasticity (GPa)	12,49

**Fig. 3:**
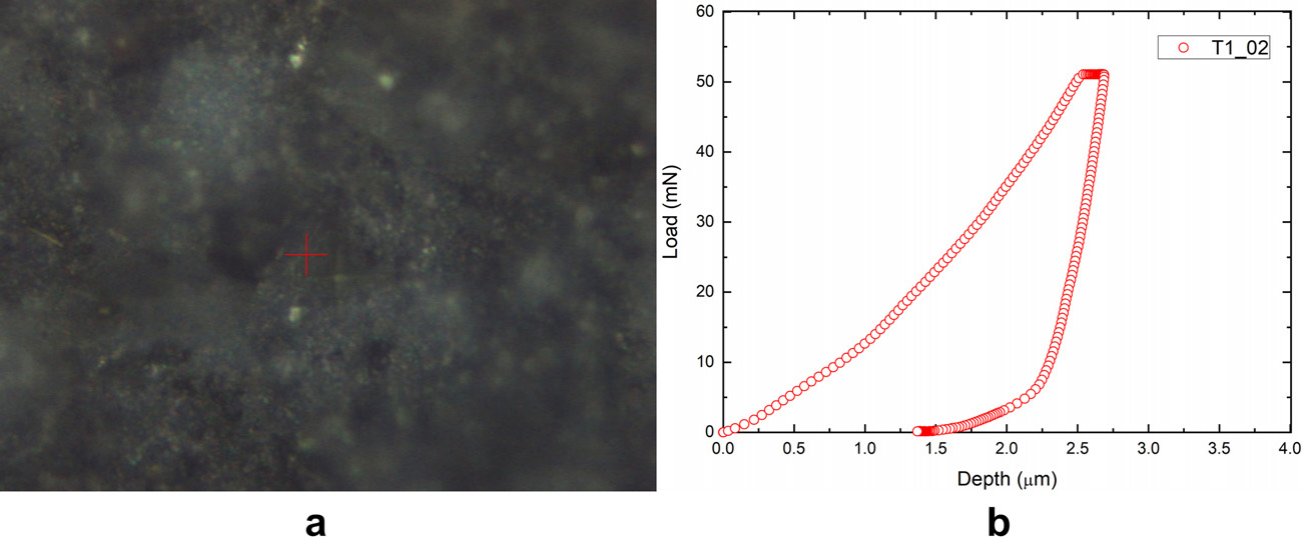
Nanoindentation test results of AAM 60B40S 0 mol/L (sample T1), surface T1_02 (a) OM image and (b) P-h curve.

The Fig. 3 shows the nanoindentation test results of AAM 60B40S 0 mol/L (sample T1), for surface T1_02. The Fig. 3(a) shows OM image and Fig. 3(b) shows P-h curve. The Table 2 shows micromechanical properties.

**Table 2:** Micromechanical properties of AAM 60B40S 0 mol/L (sample T1), surface T1_02.

Micromechanical properties	
Hardness (Hv)	54,48
Modulus of elasticity (GPa)	10,82

**Fig. 4:**
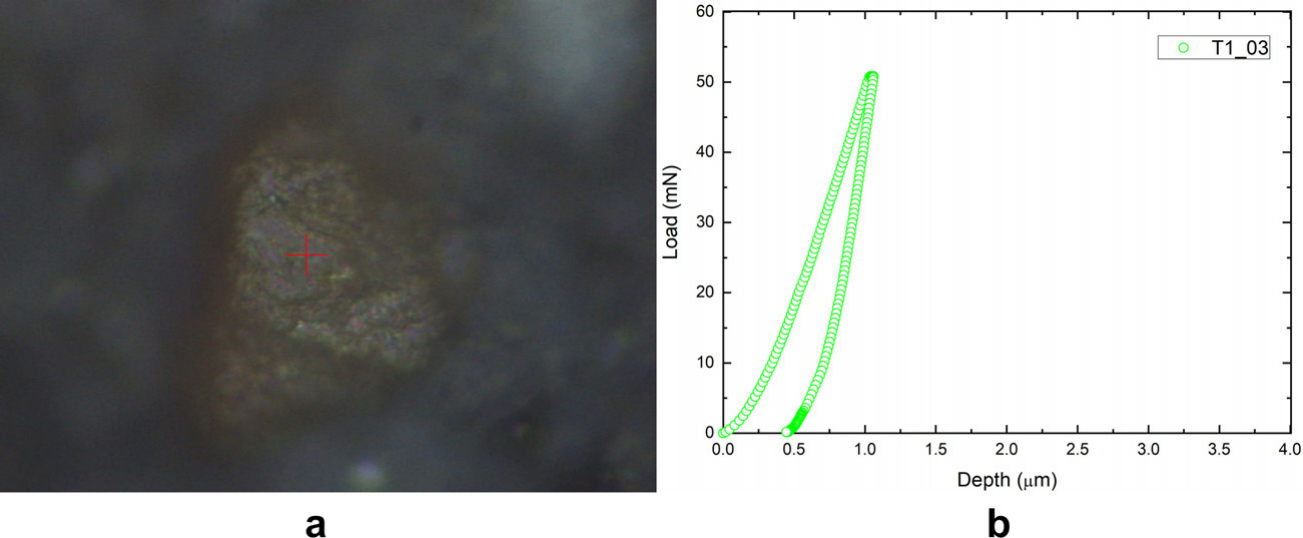
Nanoindentation test results of AAM 60B40S 0 mol/L (sample T1), surface T1_03 (a) OM image and (b) P-h curve.

The Fig. 4 shows the nanoindentation test results of AAM 60B40S 0 mol/L (sample T1), for surface T1_03. The Fig. 4(a) shows OM image and Fig. 4(b) shows P-h curve. The Table 3 shows micromechanical properties.

**Table 3:** Micromechanical properties of AAM 60B40S 0 mol/L (sample T1), surface T1_03.

Micromechanical properties	
Hardness (Hv)	432,43
Modulus of elasticity (GPa)	32,76

**Fig. 5:**
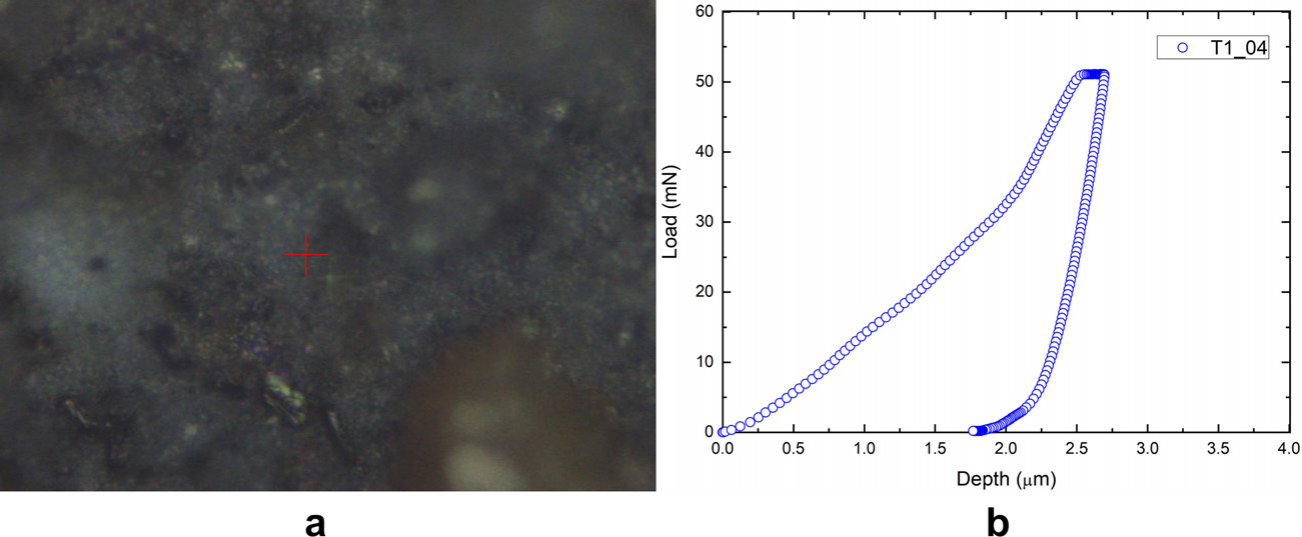
Nanoindentation test results of AAM 60B40S 0 mol/L (sample T1), surface T1_04 (a) OM image and (b) P-h curve.

The Fig. 5 shows the nanoindentation test results of AAM 60B40S 0 mol/L (sample T1), for surface T1_04. The Fig. 5(a) shows OM image and Fig. 5(b) shows P-h curve. The Table 4 shows micromechanical properties.

**Table 4:** Micromechanical properties of AAM 60B40S 0 mol/L (sample T1), surface T1_04.

Micromechanical properties	
Hardness (Hv)	244,75
Modulus of elasticity (GPa)	10,33

**Fig. 6:**
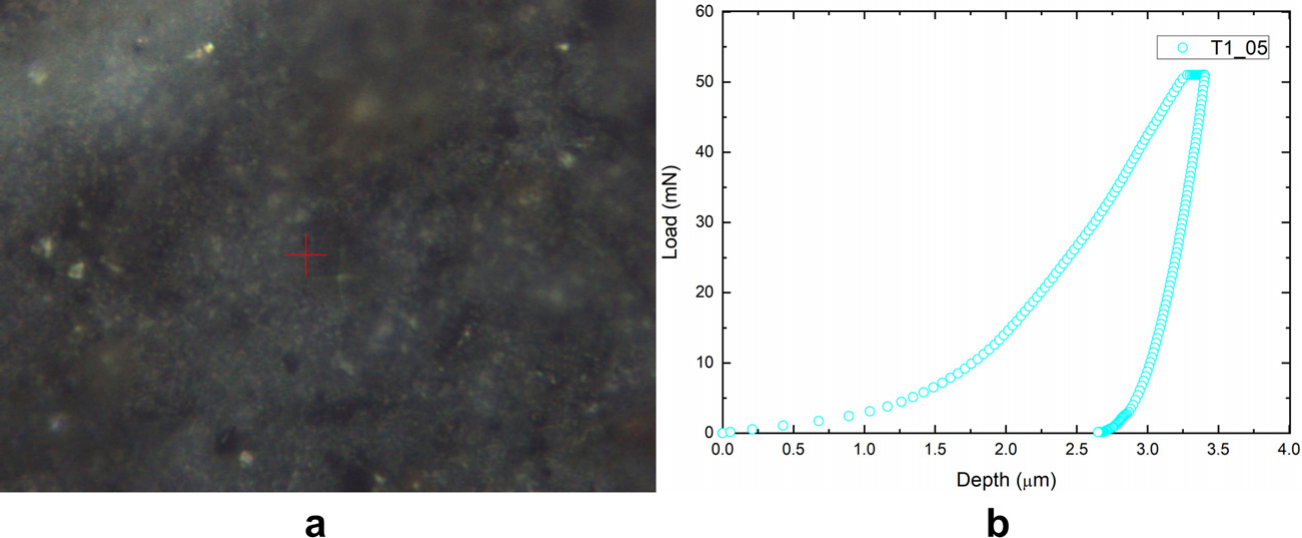
Nanoindentation test results of AAM 60B40S 0 mol/L (sample T1), surface T1_05 (a) OM image and (b) P-h curve.

The Fig. 6 shows the nanoindentation test results of AAM 60B40S 0 mol/L (sample T1), for surface T1_05. The Fig. 6(a) shows OM image and Fig. 6(b) shows P-h curves. The Table 5 shows micromechanical properties.

**Table 5:** Micromechanical properties of AAM 60B40S 0 mol/L (sample T1), surface T1_05.

Micromechanical properties	
Hardness (Hv)	119,64
Modulus of elasticity (GPa)	7,89

**Fig. 7:**
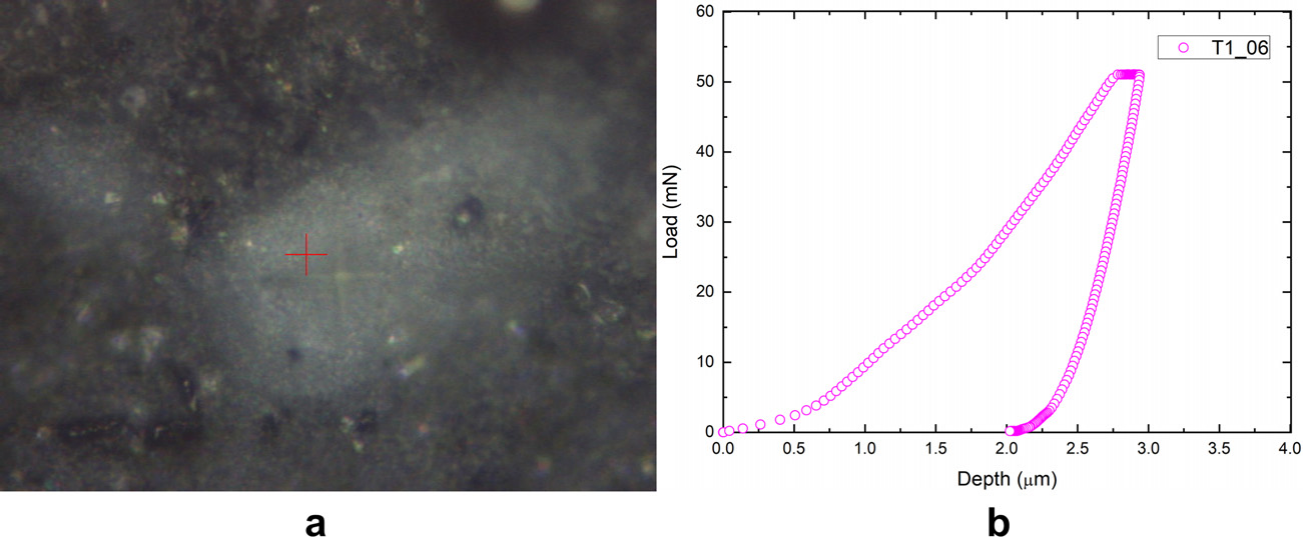
Nanoindentation test results of AAM 60B40S 0 mol/L (sample T1), surface T1_06 (a) OM image and (b) P-h curve.

The Fig. 7 shows the nanoindentation test results of AAM 60B40S 0 mol/L (sample T1), for surface T1_06. The Fig. 7(a) shows OM image and Fig. 7(b) shows P-h curve. The Table 6 shows micromechanical properties.

**Table 6:** Micromechanical properties of AAM 60B40S 0 mol/L (sample T1), surface T1_06.

Micromechanical properties	
Hardness (Hv)	37,68
Modulus of elasticity (GPa)	7,80

**Fig. 8:**
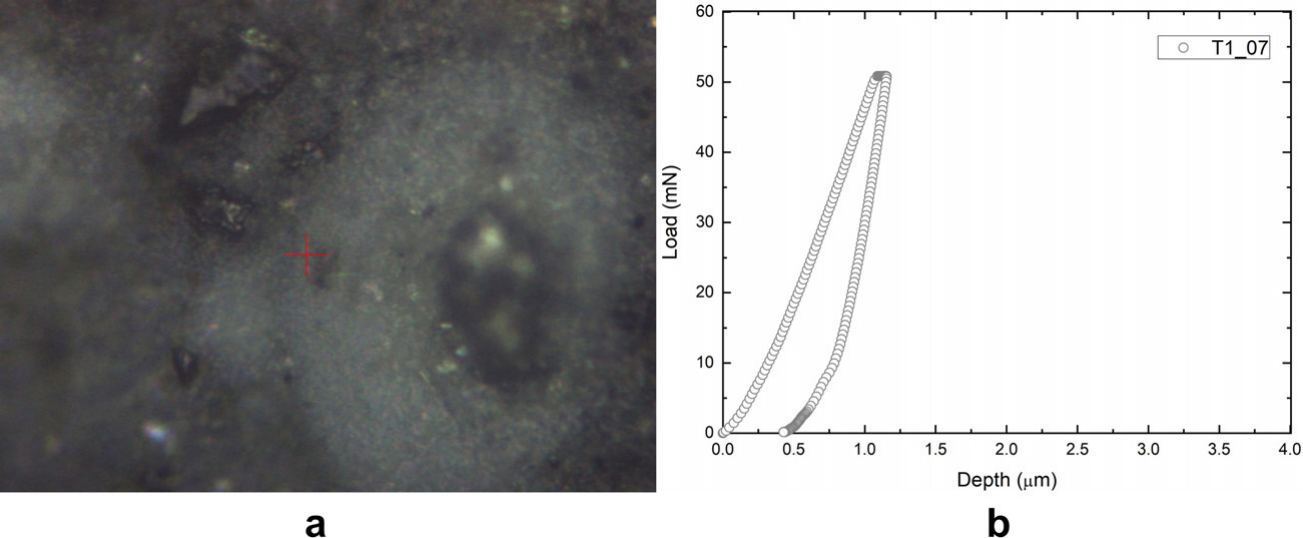
Nanoindentation test results of AAM 60B40S 0 mol/L (sample T1), surface T1_07 (a) OM image and (b) P-h curve.

The Fig. 8 shows the nanoindentation test results of AAM 60B40S 0 mol/L (sample T1), for surface T1_07. The Fig. 8(a) shows OM image and Fig. 8(b) shows P-h curve. The Table 7 shows micromechanical properties.

**Table 7:** Micromechanical properties of AAM 60B40S 0 mol/L (sample T1), surface T1_07.

Micromechanical properties	
Hardness (Hv)	168,34
Modulus of elasticity (GPa)	29,11

**Fig. 9:**
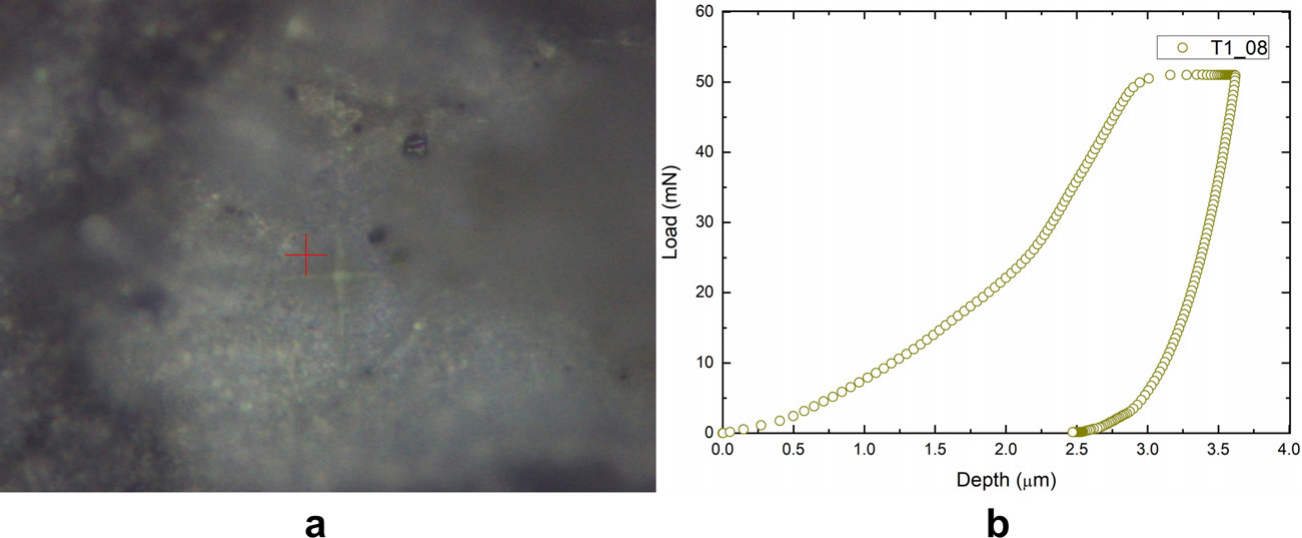
Nanoindentation test results of AAM 60B40S 0 mol/L (sample T1), surface T1_08 (a) OM image and (b) P-h curve.

The Fig. 9 shows the nanoindentation test results of AAM 60B40S 0 mol/L (sample T1), for surface T1_08. The Fig. 9(a) shows OM image and Fig. 9(b) shows P-h curve. The Table 8 shows micromechanical properties.

**Table 8:** Micromechanical properties of AAM 60B40S 0 mol/L (sample T1), surface T1_08.

Micromechanical properties	
Hardness (Hv)	34,63
Modulus of elasticity (GPa)	6,60

**Fig. 10:**
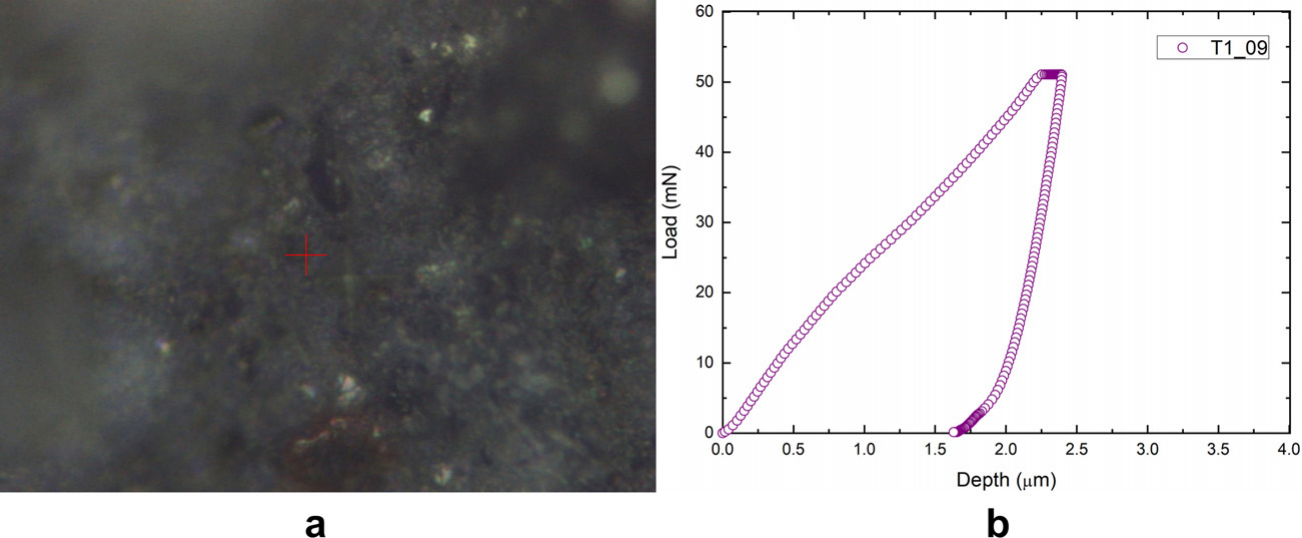
Nanoindentation test results of AAM 60B40S 0 mol/L (sample T1), surface T1_09 (a) OM image and (b) P-h curve.

The Fig. 10 shows the nanoindentation test results of AAM 60B40S 0 mol/L (sample T1), for surface T1_09. The Fig. 10(a) shows OM image and Fig. 10(b) shows P-h curve. The Table 9 shows micromechanical properties.

**Table 9:** Micromechanical properties of AAM 60B40S 0 mol/L (sample T1), surface T1_09.

Micromechanical properties	
Hardness (Hv)	44,02
Modulus of elasticity (GPa)	11,89

**Fig. 11:**
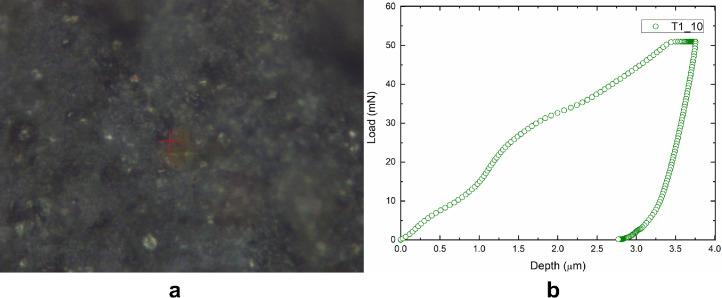
Nanoindentation test results of AAM 60B40S 0 mol/L (sample T1), surface T1_10 (a) OM image and (b) P-h curve.

The Fig. 11 shows the nanoindentation test results of AAM 60B40S 0 mol/L (sample T1), for surface T1_10. The Fig. 11(a) shows OM image and Fig. 11(b) shows P-h curve. The Table 10 shows micromechanical properties.

**Table 10:** Micromechanical properties of AAM 60B40S 0 mol/L (sample T1), surface T1_10.

Micromechanical properties	
Hardness (Hv)	65,66
Modulus of elasticity (GPa)	6,17

**Fig. 12:**
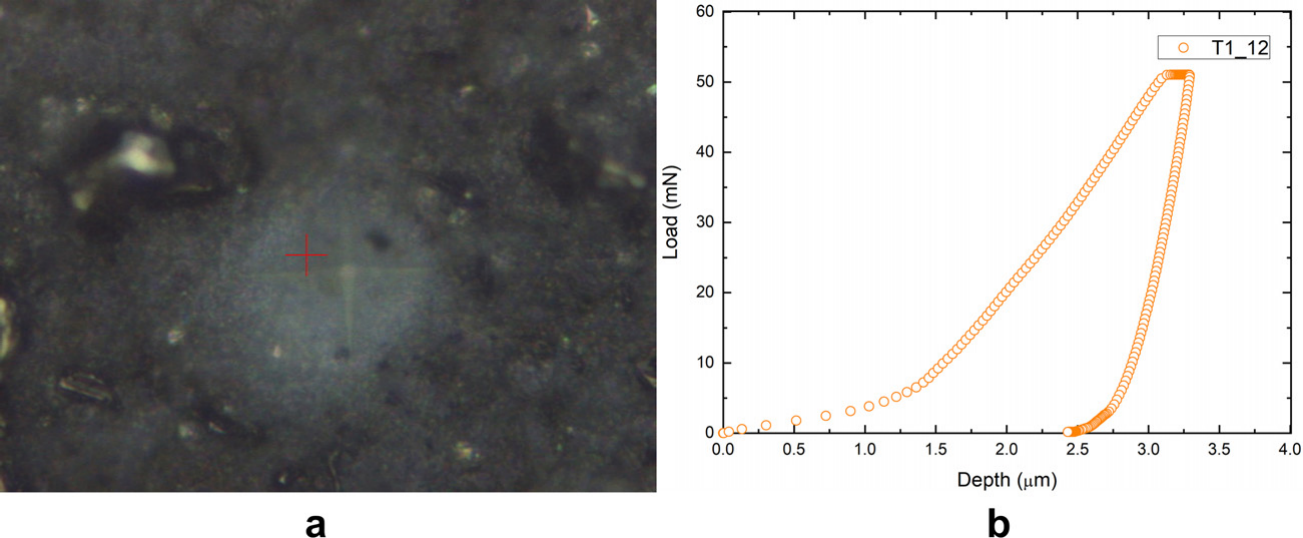
Nanoindentation test results of AAM 60B40S 0 mol/L (sample T1), surface T1_12 (a) OM image and (b) P-h curve.

The Fig. 12 shows the nanoindentation test results of AAM 60B40S 0 mol/L (sample T1), for surface T1_12. The Fig. 12(a) shows OM image and Fig. 12(b) shows P-h curve. The Table 11 shows micromechanical properties.

**Table 11:** Micromechanical properties of AAM 60B40S 0 mol/L (sample T1), surface T1_12.

Micromechanical properties	
Hardness (Hv)	10,41
Modulus of elasticity (GPa)	7,83

**Fig. 13:**
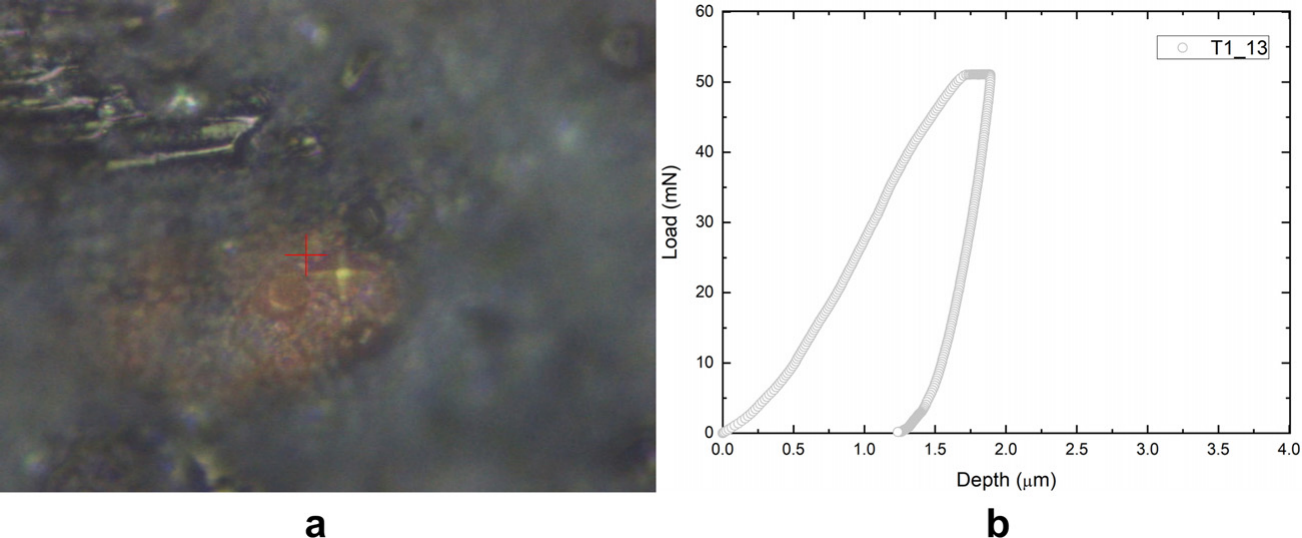
Nanoindentation test results of AAM 60B40S 0 mol/L (sample T1), surface T1_13 (a) OM image and (b) P-h curve.

The Fig. 13 shows the nanoindentation test results of AAM 60B40S 0 mol/L (sample T1), for surface T1_13. The Fig. 13(a) shows OM image and Fig. 13(b) shows P-h curve. The Table 12 shows micromechanical properties.

**Table 12:** Micromechanical properties of AAM 60B40S 0 mol/L (sample T1), surface T1_13.

Micromechanical properties	
Hardness (Hv)	78,79
Modulus of elasticity (GPa)	18,14

**Fig. 14:**
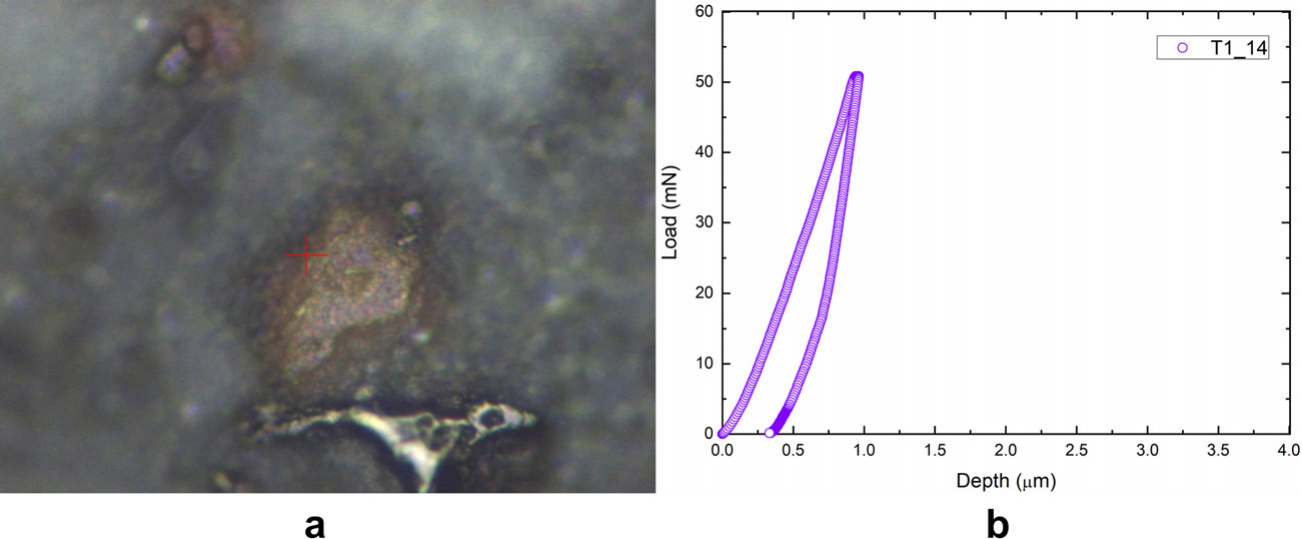
Nanoindentation test results of AAM 60B40S 0 mol/L (sample T1), surface T1_14 (a) OM image and (b) P-h curve.

The Fig. 14 shows the nanoindentation test results of AAM 60B40S 0 mol/L (sample T1), for surface T1_14. The Fig. 14(a) shows OM image and Fig. 14(b) shows P-h curve. The Table 13 shows micromechanical properties.

**Table 13:** Micromechanical properties of AAM 60B40S 0 mol/L (sample T1), surface T1_14.

Micromechanical properties	
Hardness (Hv)	297,09
Modulus of elasticity (GPa)	39,21

#### 2.1.2 AAM 60B40S 5 mol/L (sample T4)

**Fig. 15:**
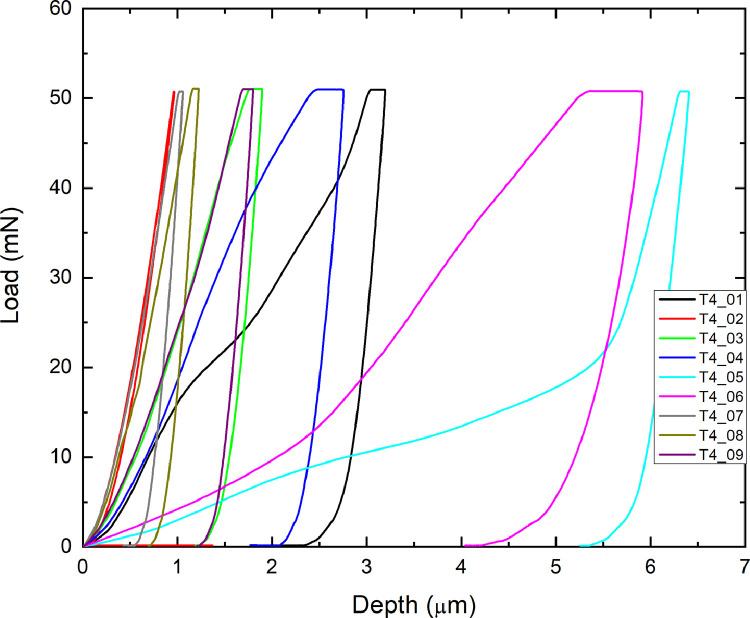
P-h curves for sample T4.

The Fig. 15 shows the load-depth (P-h) curves for sample T4 (60B40S 5 mol/L), grouped in a single graph.

**Fig. 16:**
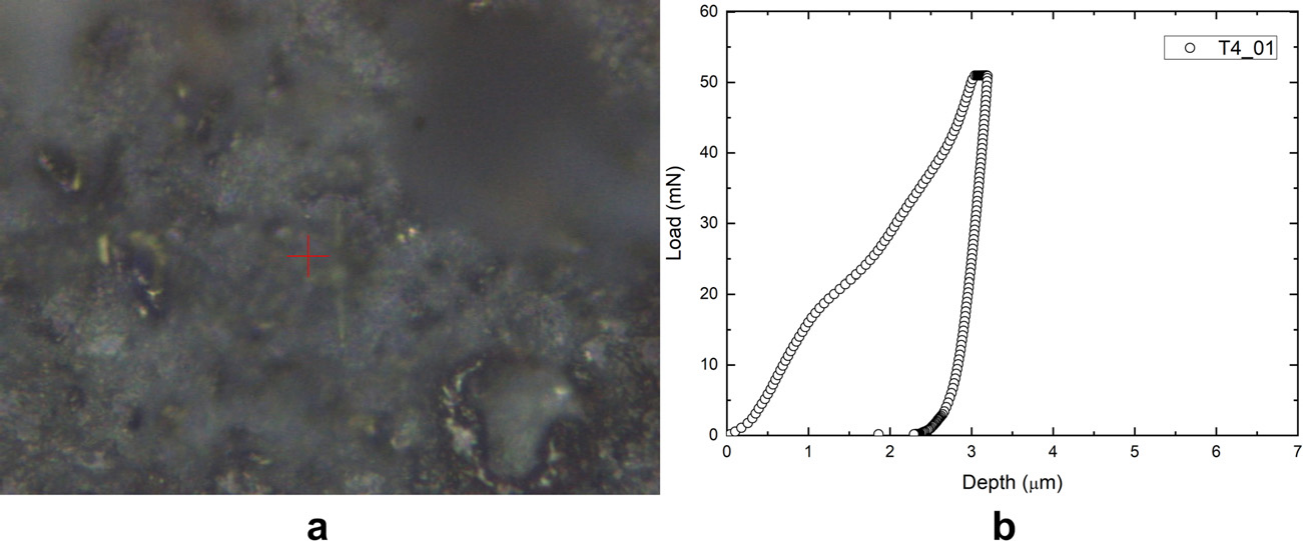
Nanoindentation test results of AAM 60B40S 5 mol/L (sample T4), surface T4_01 (a) OM image and (b) P-h curve.

The Fig. 16 shows the nanoindentation test results of AAM 60B40S 5 mol/L (sample T4), for surface T4_01. The Fig. 16(a) shows OM image and Fig. 16(b) shows P-h curve. The Table 14 shows micromechanical properties.

**Table 14:** Micromechanical properties of AAM 60B40S 5 mol/L (sample T4), surface T4_01.

Micromechanical properties	
Hardness (Hv)	22,59
Modulus of elasticity (GPa)	8,89

**Fig. 17:**
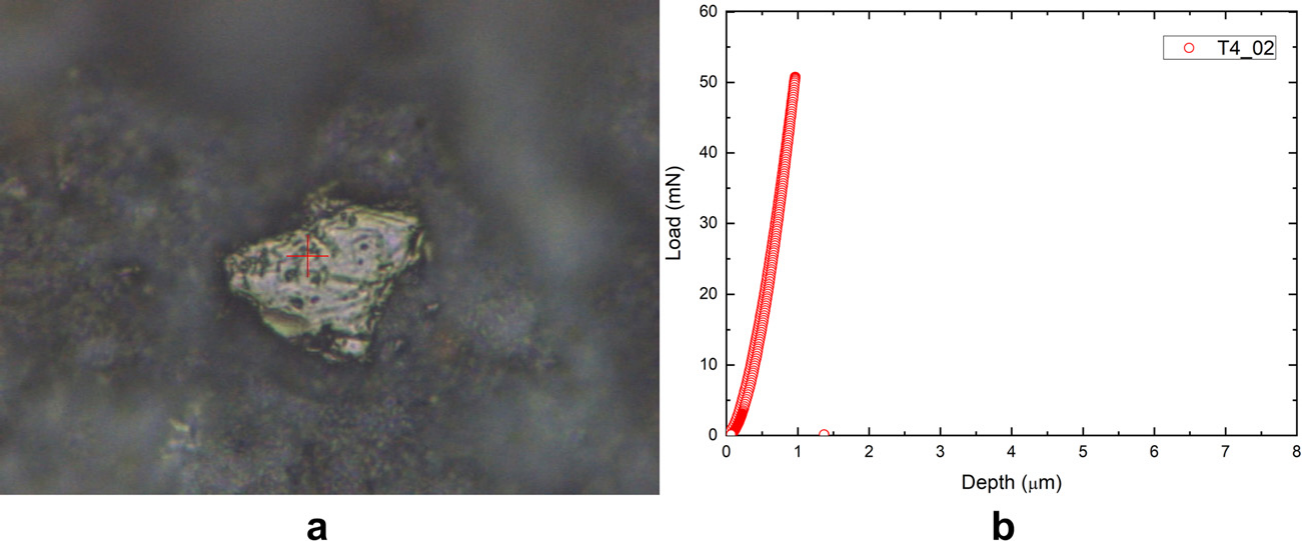
Nanoindentation test results of AAM 60B40S 5 mol/L (sample T4), surface T4_02 (a) OM image and (b) P-h curve.

The Fig. 17 shows the nanoindentation test results of AAM 60B40S 5 mol/L (sample T4), for surface T4_02. The Fig. 17(a) shows OM image and Fig. 17(b) shows P-h curve. The Table 15 shows micromechanical properties.

**Table 15:** Micromechanical properties of AAM 60B40S 5 mol/L (sample T4), surface T4_02.

Micromechanical properties	
Hardness (Hv)	-
Modulus of elasticity (GPa)	30,05

**Fig. 18:**
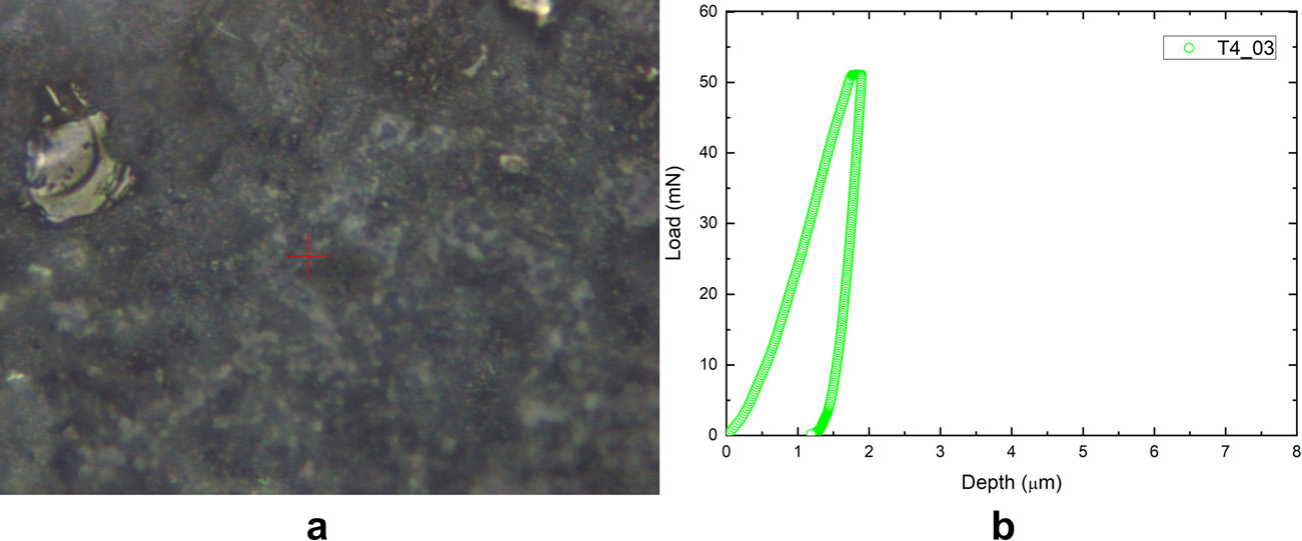
Nanoindentation test results of AAM 60B40S 5 mol/L (sample T4), surface T4_03 (a) OM image and (b) P-h curve.

The Fig. 18 shows the nanoindentation test results of AAM 60B40S 5 mol/L (sample T4), for surface T4_03. The Fig. 18(a) shows OM image and Fig. 18(b) shows P-h curve. The Table 16 shows micromechanical properties.

**Table 16:** Micromechanical properties of AAM 60B40S 5 mol/L (sample T4), surface T4_03.

Micromechanical properties	
Hardness (Hv)	54,45
Modulus of elasticity (GPa)	16,88

**Fig. 19:**
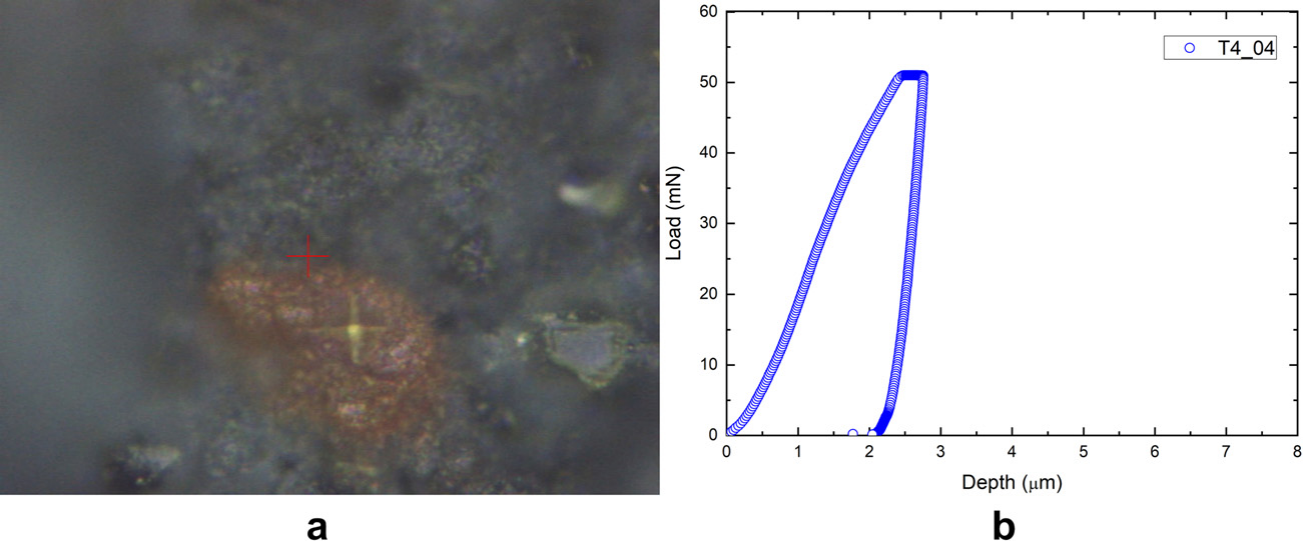
Nanoindentation test results of AAM 60B40S 5 mol/L (sample T4), surface T4_04 (a) OM image and (b) P-h curve.

The Fig. 19 shows the nanoindentation test results of AAM 60B40S 5 mol/L (sample T4), for surface T4_04. The Fig. 19(a) shows OM image and Fig. 19(b) shows P-h curve. The Table 17 shows micromechanical properties.

**Table 17:** Micromechanical properties of AAM 60B40S 5 mol/L (sample T4), surface T4_04.

Micromechanical properties	
Hardness (Hv)	81,93
Modulus of elasticity (GPa)	10,62

**Fig. 20:**
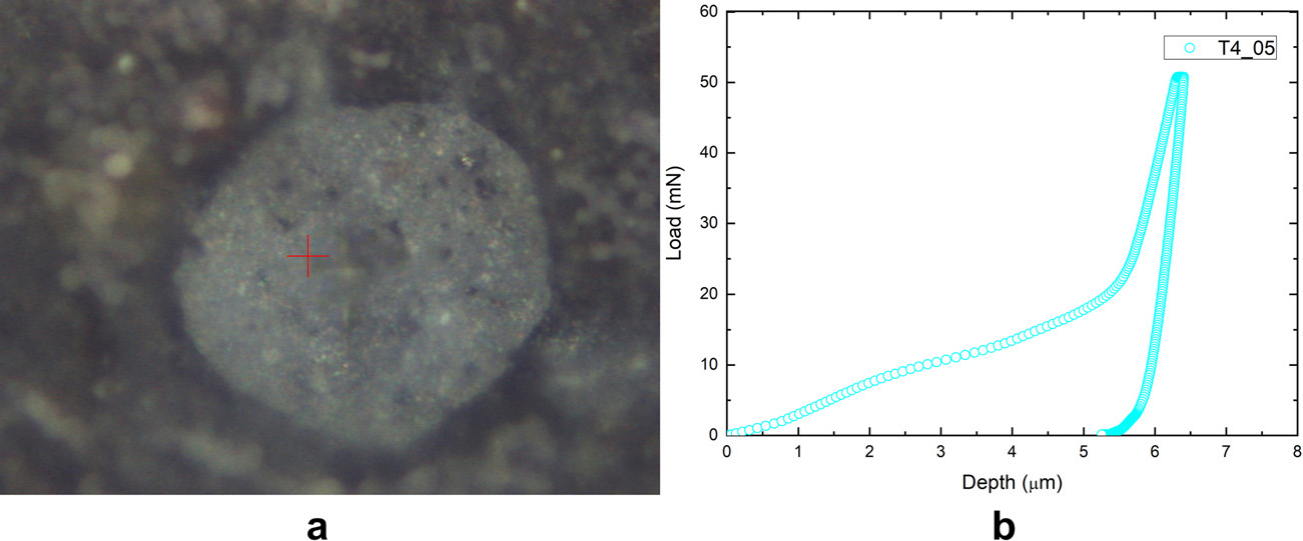
Nanoindentation test results of AAM 60B40S 5 mol/L (sample T4), surface T4_05 (a) OM image and (b) P-h curve.

The Fig. 20 shows the nanoindentation test results of AAM 60B40S 5 mol/L (sample T4), for surface T4_05. The Fig. 20(a) shows OM image and Fig. 20(b) shows P-h curve. The Table 18 shows micromechanical properties.

**Table 18:** Micromechanical properties of AAM 60B40S 5 mol/L (sample T4), surface T4_05.

Micromechanical properties	
Hardness (Hv)	71,29
Modulus of elasticity (GPa)	3,28

**Fig. 21:**
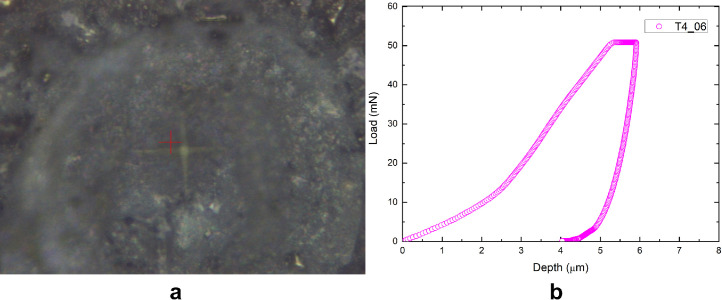
Nanoindentation test results of AAM 60B40S 5 mol/L (sample T4), surface T4_06 (a) OM image and (b) P-h curve.

The Fig. 21 shows the nanoindentation test results of AAM 60B40S 5 mol/L (sample T4), for surface T4_06. The Fig. 21(a) shows OM image and Fig. 21(b) shows P-h curve. The Table 19 shows micromechanical properties.

**Table 19:** Micromechanical properties of AAM 60B40S 5 mol/L (sample T4), surface T4_06.

Micromechanical properties	
Hardness (Hv)	17,63
Modulus of elasticity (GPa)	3,07

**Fig. 22:**
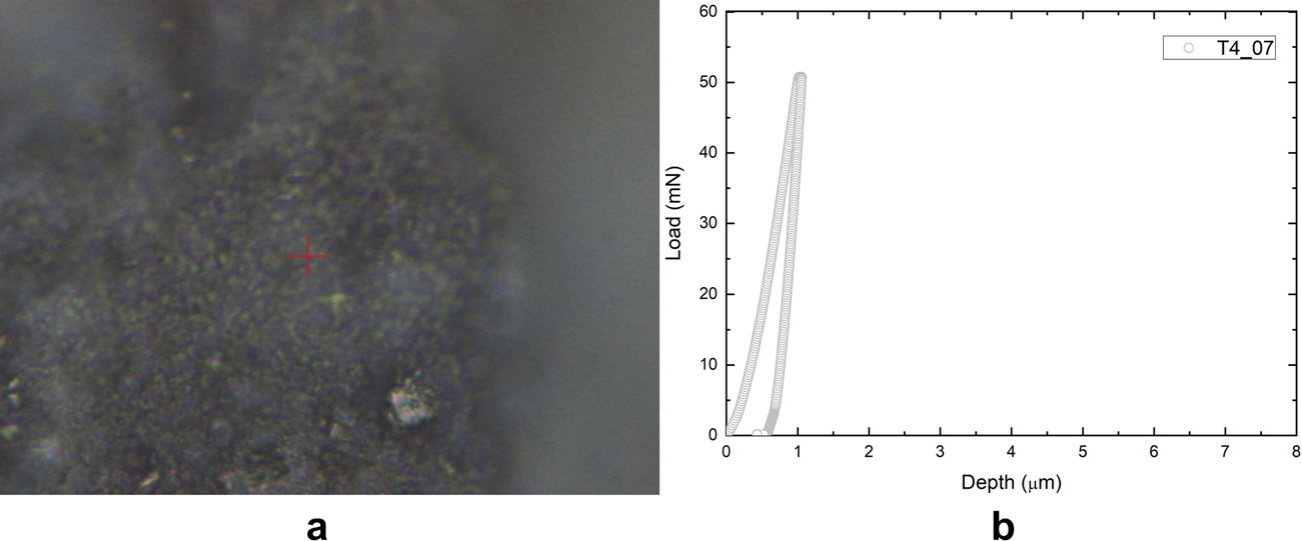
Nanoindentation test results of AAM 60B40S 5 mol/L (sample T4), surface T4_07 (a) OM image and (b) P-h curve.

The Fig. 22 shows the nanoindentation test results of AAM 60B40S 5 mol/L (sample T4), for surface T4_07. The Fig. 22(a) shows OM image and Fig. 22(b) shows P-h curve. The Table 20 shows micromechanical properties.

**Table 20:** Micromechanical properties of AAM 60B40S 5 mol/L (sample T4), surface T4_07.

Micromechanical properties	
Hardness (Hv)	185,99
Modulus of elasticity (GPa)	36,51

**Fig. 23:**
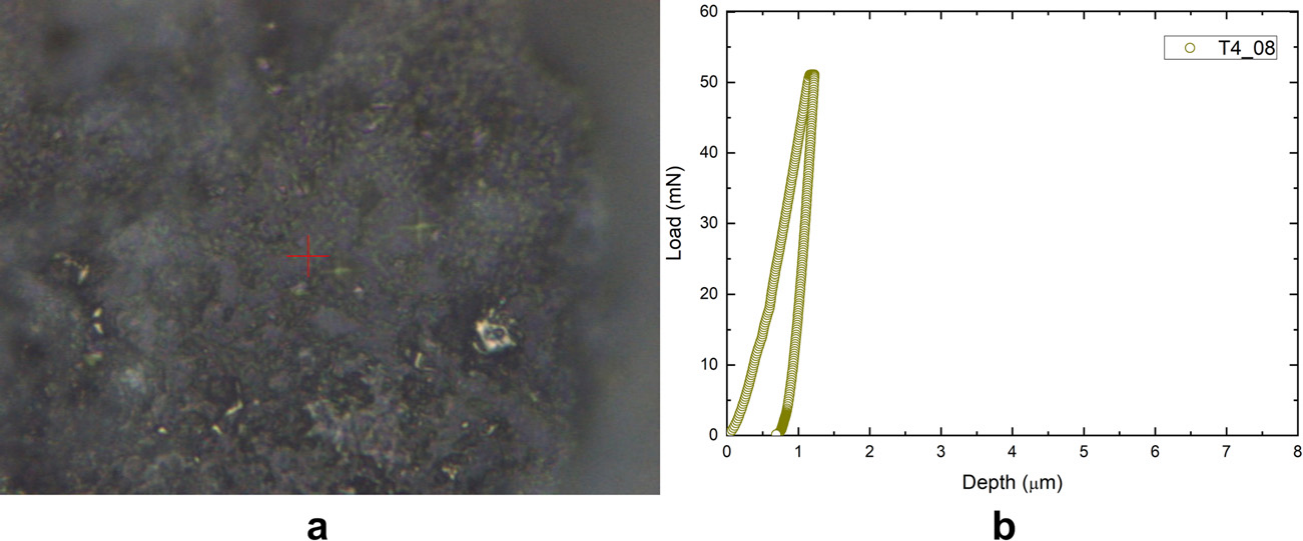
Nanoindentation test results of AAM 60B40S 5 mol/L (sample T4), surface T4_08 (a) OM image and (b) P-h curve.

The Fig. 23 shows the nanoindentation test results of AAM 60B40S 5 mol/L (sample T4), for surface T4_08. The Fig. 23(a) shows OM image and Fig. 23(b) shows P-h curve. The Table 21 shows micromechanical properties.

**Table 21:** Micromechanical properties of AAM 60B40S 5 mol/L (sample T4), surface T4_08.

Micromechanical properties	
Hardness (Hv)	222,93
Modulus of elasticity (GPa)	30,95

**Fig. 24:**
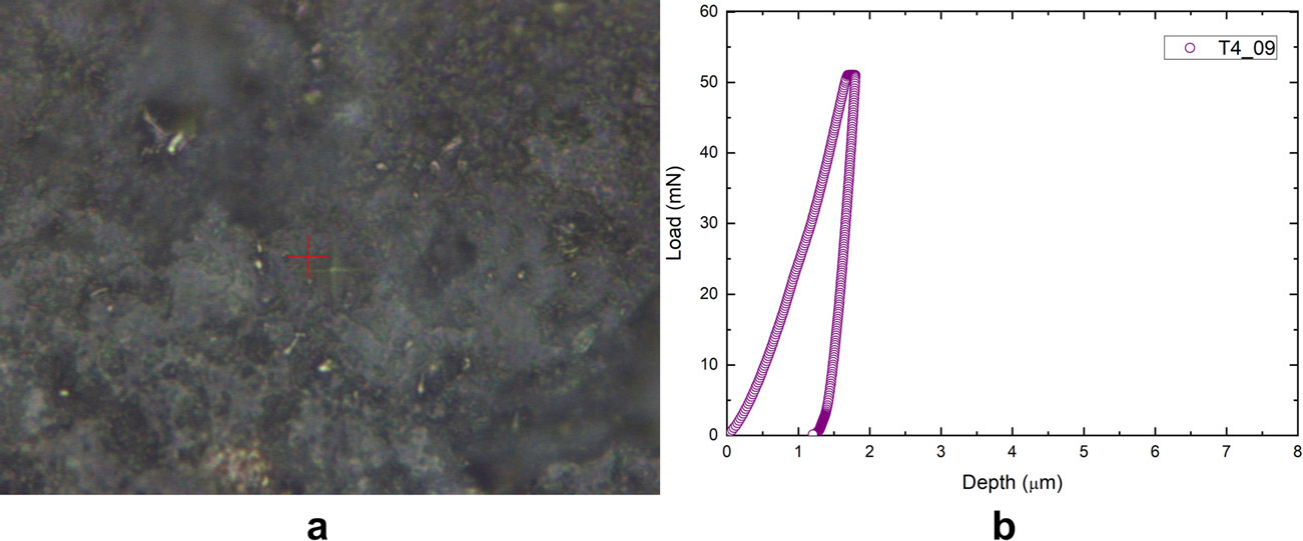
Nanoindentation test results of AAM 60B40S 5 mol/L (sample T4), surface T4_09 (a) OM image and (b) P-h curve.

The Fig. 24 shows the nanoindentation test results of AAM 60B40S 5 mol/L (sample T4), for surface T4_09. The Fig. 24(a) shows OM image and Fig. 24(b) shows P-h curve. The Table 22 shows micromechanical properties.

**Table 22:** Micromechanical properties of AAM 60B40S 5 mol/L (sample T4), surface T4_09.

Micromechanical properties	
Hardness (Hv)	121,76
Modulus of elasticity (GPa)	18,75

#### 2.1.3 AAM 50B50S 5 mol/L (sample T5)

**Fig. 25:**
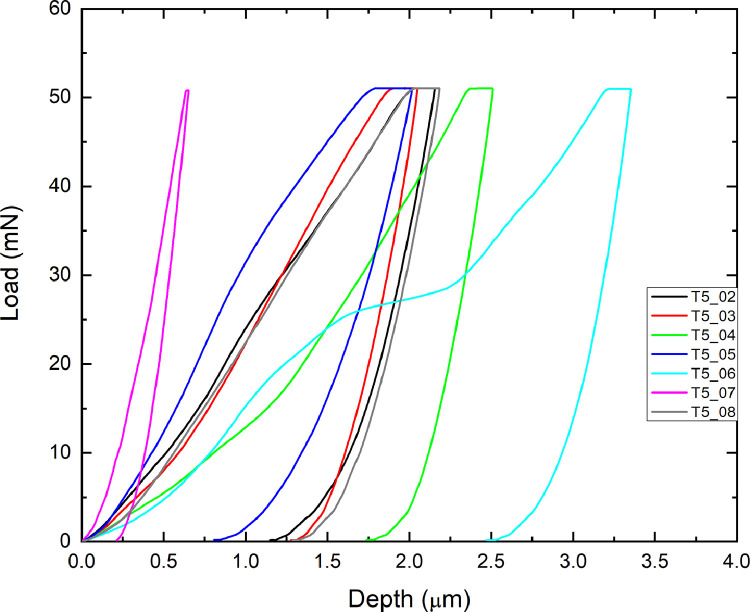
P-h for sample T5.

A Fig. 25 shows the load-depth (P-h) curves for sample T5 (50B50S 5 mol/L), grouped in a single graph.

**Fig. 26:**
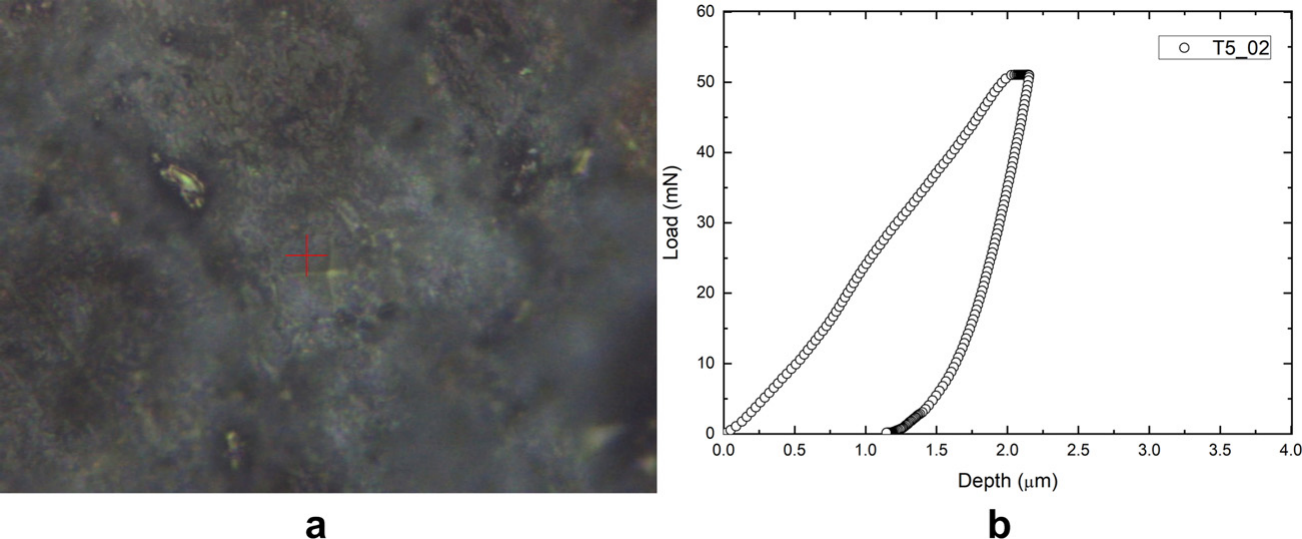
Nanoindentation test results of AAM 50B50S 5 mol/L (sample T5), surface T5_02 (a) OM image and (b) P-h curve.

The Fig. 26 shows the nanoindentation test results of AAM 50B50S 5 mol/L (sample T5), for surface T5_02. The Fig. 26(a) shows OM image and Fig. 26(b) shows P-h curve. The Table 23 shows micromechanical properties.

**Table 23:** Micromechanical properties of AAM 50B50S 5 mol/L (sample T5), surface T5_02.

Micromechanical properties	
Hardness (Hv)	103,54
Modulus of elasticity (GPa)	10,51

**Fig. 27:**
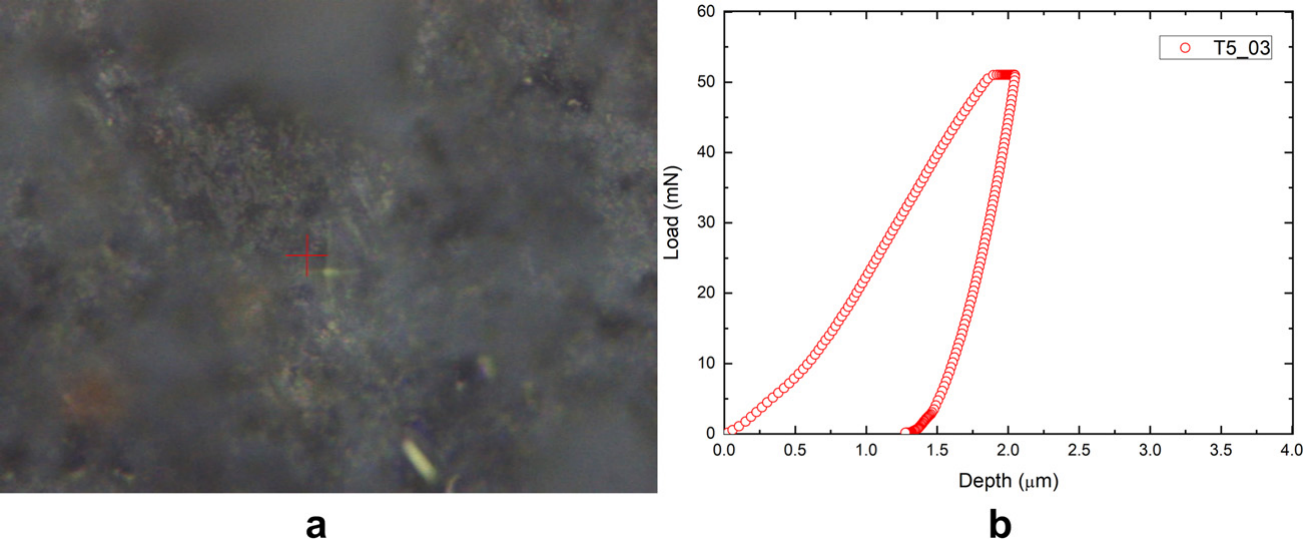
Nanoindentation test results of AAM 50B50S 5 mol/L (sample T5), surface T5_03 (a) OM image and (b) P-h curve.

The Fig. 27 shows the nanoindentation test results of AAM 50CCE50S 5 mol/L (sample T5), for surface T5_03. The Fig. 27(a) shows OM image and Fig. 27(b) shows P-h curve. The Table 24 shows micromechanical properties.

**Table 24:** Micromechanical properties of AAM 50B50S 5 mol/L (sample T5), surface T5_03.

Micromechanical properties	
Hardness (Hv)	93,16
Modulus of elasticity (GPa)	12,48

**Fig. 28:**
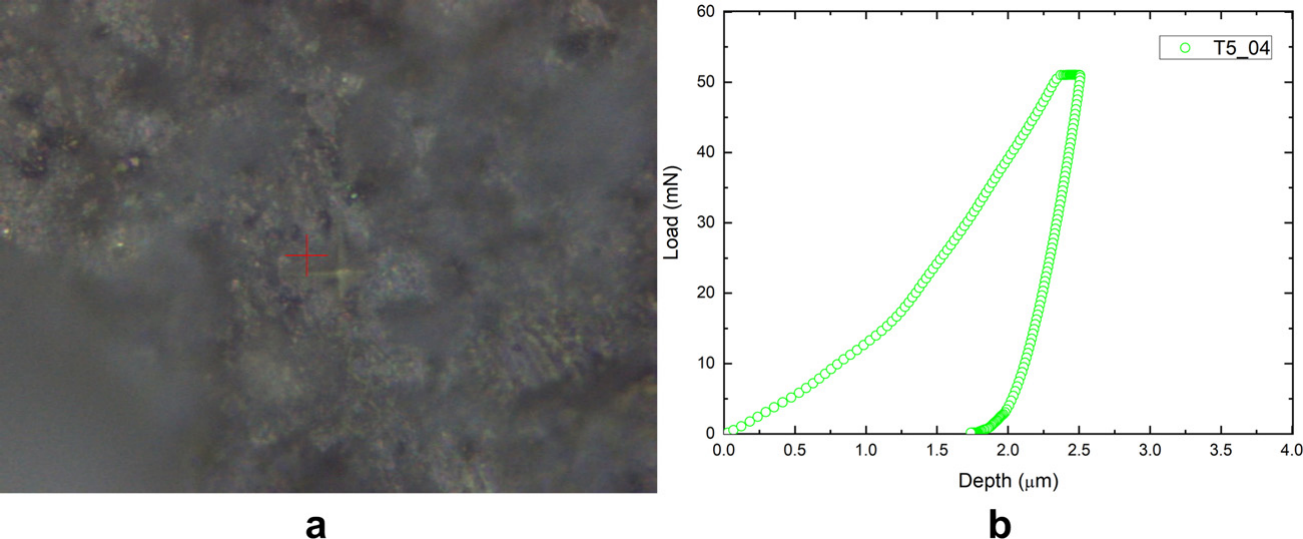
Nanoindentation test results of AAM 50B50S 5 mol/L (sample T5), surface T5_04 (a) OM image and (b) P-h curve.

The Fig. 28 shows the nanoindentation test results of AAM 50B50S 5 mol/L (sample T5), for surface T5_04. The Fig. 28(a) shows OM image and Fig. 28(b) shows P-h curve. The Table 25 shows micromechanical properties.

**Table 25:** Micromechanical properties of AAM 50B50S 5 mol/L (sample T5), surface T5_05.

Micromechanical properties	
Hardness (Hv)	85,47
Modulus of elasticity (GPa)	10,64

**Fig. 29:**
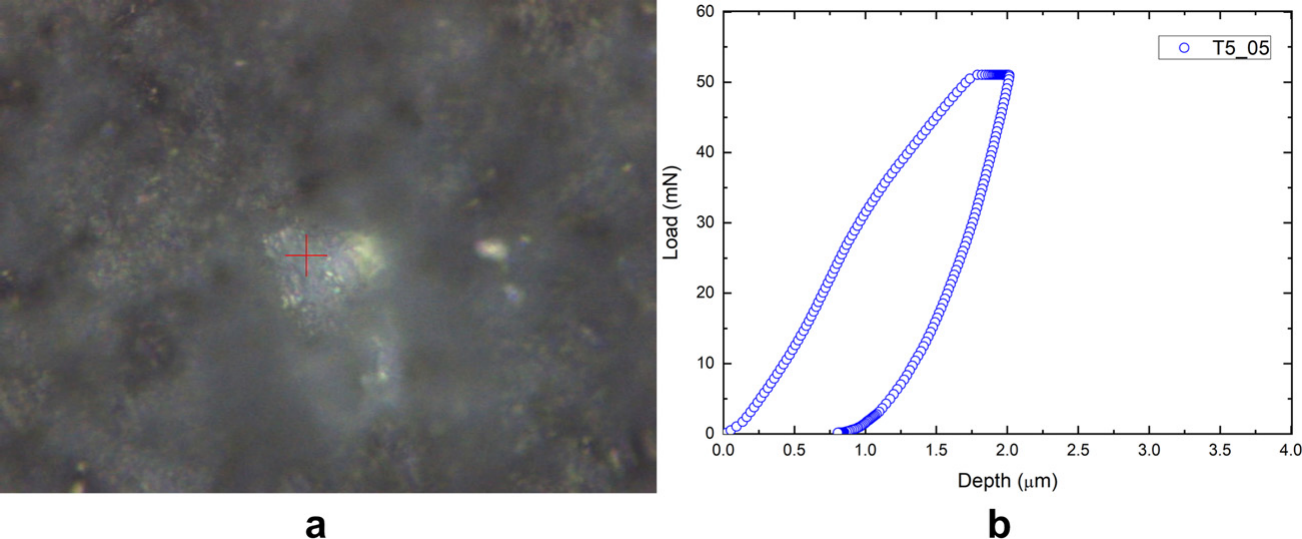
Nanoindentation test results of AAM 50B50S 5 mol/L (sample T5), surface T5_05 (a) OM image and (b) P-h curve.

The Fig. 29 shows the nanoindentation test results of AAM 50B50S 5 mol/L (sample T5), for surface T5_05. The Fig. 29(a) shows OM image and Fig. 29(b) shows P-h curve. The Table 26 shows micromechanical properties.

**Table 26:** Micromechanical properties of AAM 50B50S 5 mol/L (sample T5), surface T5_05.

Micromechanical properties	
Hardness (Hv)	-
Modulus of elasticity (GPa)	9,72

**Fig. 30:**
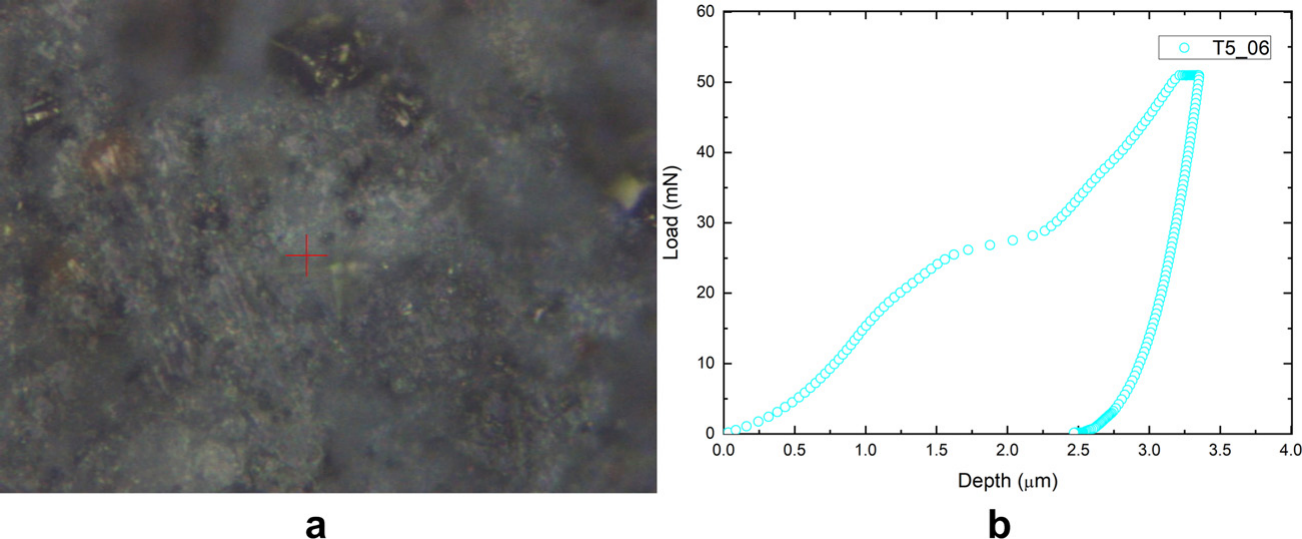
Nanoindentation test results of AAM 50B50S 5 mol/L (sample T5), surface T5_06 (a) OM image and (b) P-h curve.

The Fig. 30 shows the nanoindentation test results of AAM 50B50S 5 mol/L (sample T5), for surface T5_06. The Fig. 30(a) shows OM image and Fig. 30(b) shows P-h curve. The Table 27 shows micromechanical properties.

**Table 27:** Micromechanical properties of AAM 50B50S 5 mol/L (sample T5), surface T5_06.

Micromechanical properties	
Hardness (Hv)	78,60
Modulus of elasticity (GPa)	7,97

**Fig. 31:**
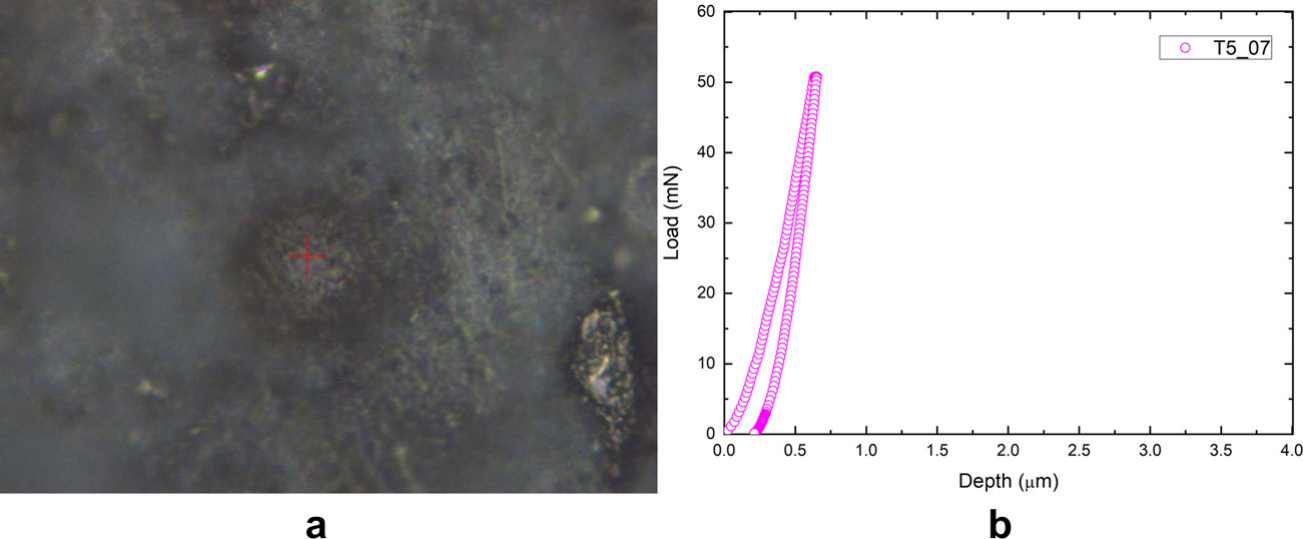
Nanoindentation test results of AAM 50B50S 5 mol/L (sample T5), surface T5_07 (a) OM image and (b) P-h curve.

The Fig. 31 shows the nanoindentation test results of AAM 50B50S 5 mol/L (sample T5), for surface T5_07. The Fig. 31(a) shows OM image and Fig. 31(b) shows P-h curve. The Table 28 shows micromechanical properties.

**Table 28:** Micromechanical properties of AAM 50B50S 5 mol/L (sample T5), surface T5_07.

Micromechanical properties	
Hardness (Hv)	744,78
Modulus of elasticity (GPa)	78,62

**Fig. 32:**
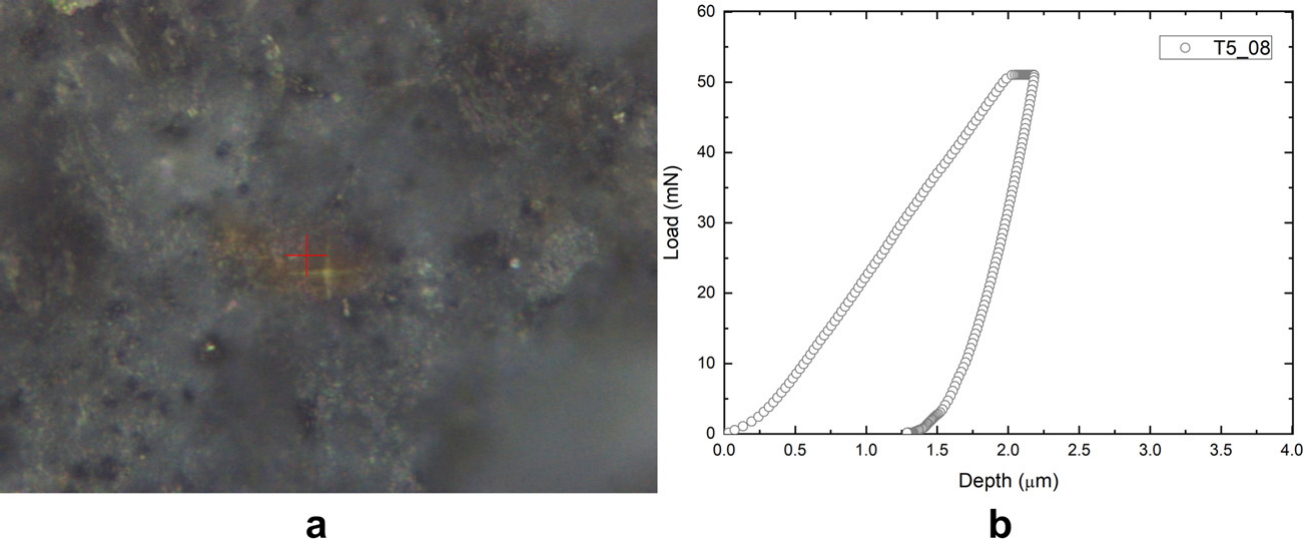
Nanoindentation test results of AAM 50B50S 5 mol/L (sample T5), surface T5_08 (a) OM image and (b) P-h curve.

The Fig. 32 shows the nanoindentation test results of AAM 50B50S 5 mol/L (sample T5), for surface T5_08. The Fig. 32(a) shows OM image and Fig. 32(b) shows P-h curve. The Table 29 shows micromechanical properties.

**Table 29:** Micromechanical properties of AAM 50B50S 5 mol/L (sample T5), surface T5_08.

Micromechanical properties	
Hardness (Hv)	123,91
Modulus of elasticity (GPa)	10,50

#### 2.1.4 AAM 40B60S 5 mol/L (sample T6)

**Fig. 33:**
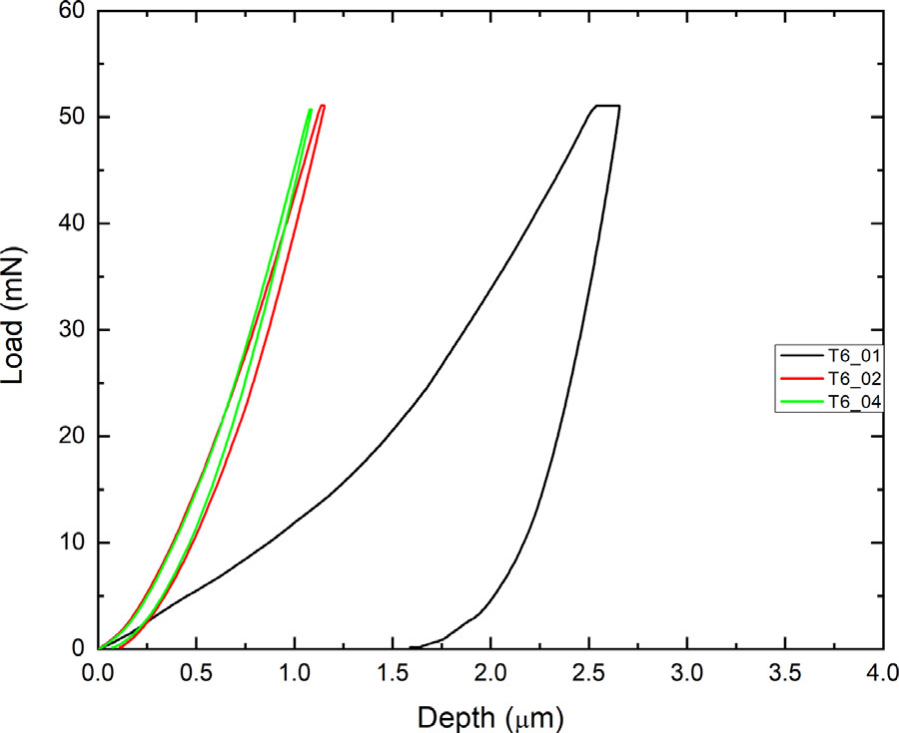
P-h curves for sample T6.

The Fig. 33 shows the load-depth (P-h) curves for sample T6 (40B60S 5 mol/L), grouped in a single graph.

**Fig. 34:**
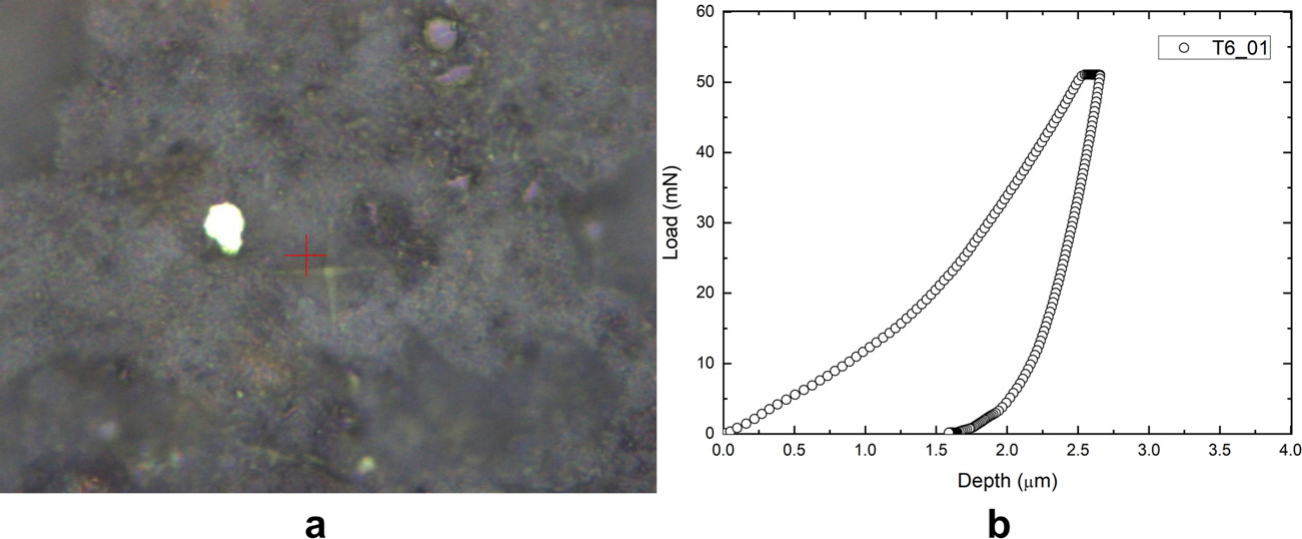
Nanoindentation test results of AAM 40B60S 5 mol/L (sample T6), surface T6_01 (a) OM image and (b) P-h curve.

The Fig. 34 shows the nanoindentation test results of AAM 40B60S 5 mol/L (sample T6), for surface T6_01. The Fig. 34(a) shows OM image and Fig. 34(b) shows P-h curve. The Table 30 shows micromechanical properties.

**Table 30:** Micromechanical properties of AAM 40B60S 5 mol/L (sample T6), surface T6_01.

Micromechanical properties	
Hardness (Hv)	56,95
Modulus of elasticity (GPa)	8,80

**Fig. 35:**
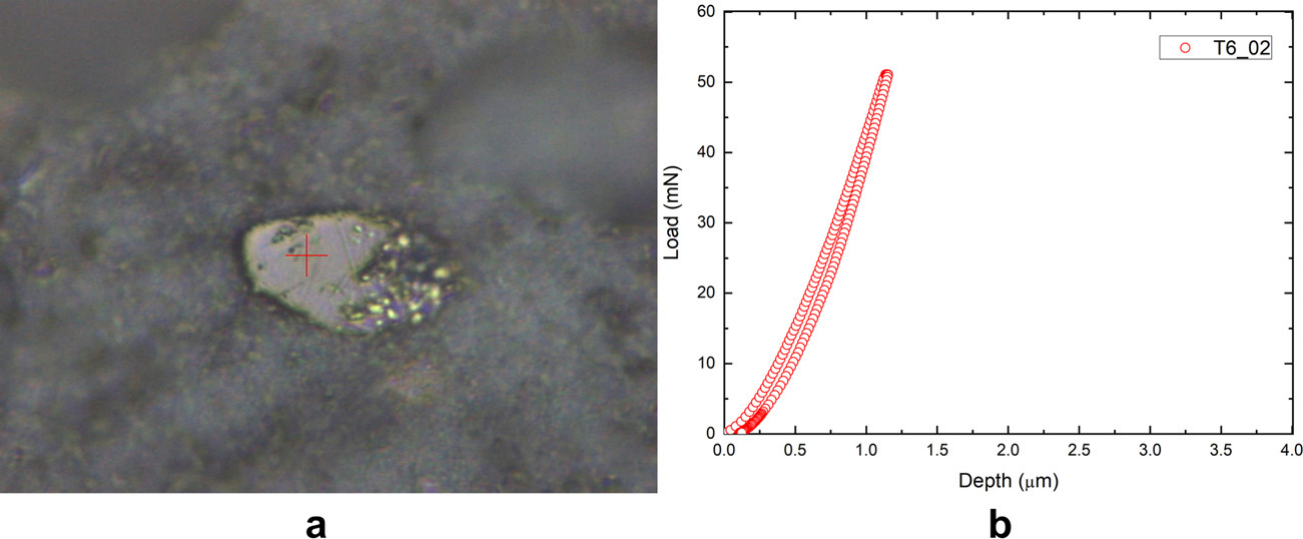
Nanoindentation test results of AAM 40B60S 5 mol/L (sample T6), surface T6_02 (a) OM image and (b) P-h curve.

The Fig. 35 shows the nanoindentation test results of AAM 40B60S 5 mol/L (sample T6), for surface T6_02. The Fig. 35(a) shows OM image and Fig. 35(b) shows P-h curve. The Table 31 shows micromechanical properties.

**Table 31:** Micromechanical properties of AAM 40B60S 5 mol/L (sample T6), surface T6_02.

Micromechanical properties	
Hardness (Hv)	-
Modulus of elasticity (GPa)	21,28

**Fig. 36:**
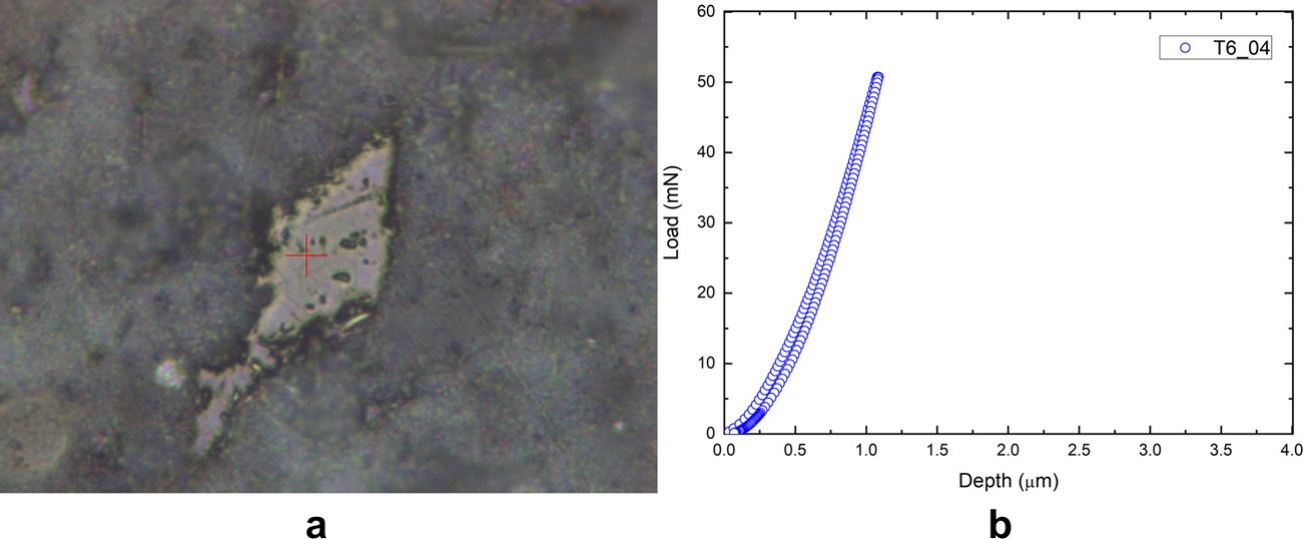
Nanoindentation test results of AAM 40B60S 5 mol/L (sample T6), surface T6_04 (a) OM image and (b) P-h curve.

The Fig. 36 shows the nanoindentation test results of AAM 40B60S 5 mol/L (sample T6), for surface T6_04. The Fig. 36(a) shows OM image and Fig. 36(b) shows P-h curve. The Table 32 shows micromechanical properties.

**Table 32:** Micromechanical properties of AAM 40B60S 5 mol/L (sample T6), surface T6_04.

Micromechanical properties	
Hardness (Hv)	-
Modulus of elasticity (GPa)	23,84

#### 2.1.5 AAM 60B40S 10 mol/L (sample T7)

**Fig. 37:**
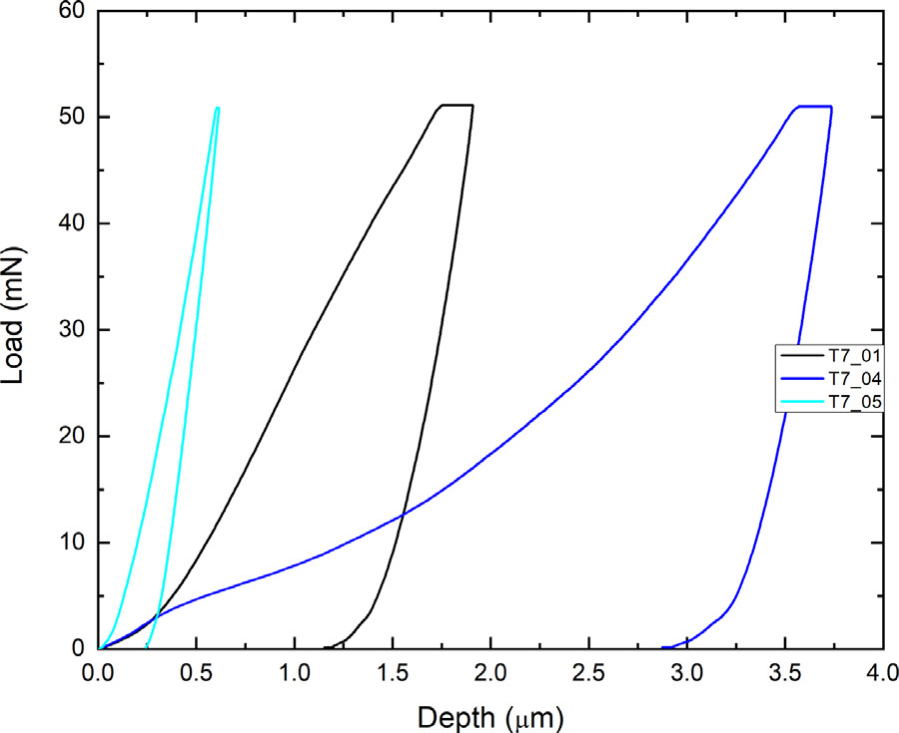
P-h curves for sample T7.

The Fig. 37 shows the load-depth (P-h) curves for sample T7 (60B40S 10 mol/L), grouped in a single graph.

**Fig. 38:**
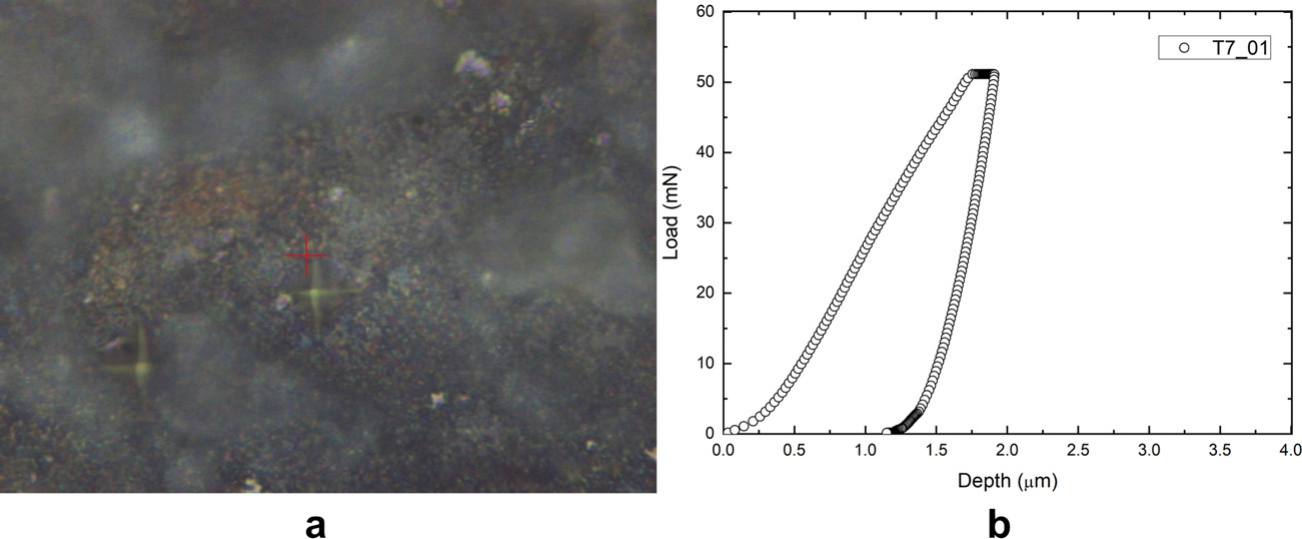
Nanoindentation test results of AAM 60B40S 10 mol/L (sample T7), surface T7_01 (a) OM image and (b) P-h curve.

The Fig. 38 shows the nanoindentation test results of AAM 60B40S 10 mol/L (sample T7), for surface T7_01. The Fig. 38(a) shows OM image and Fig. 38(b) shows P-h curve. The Table 33 shows micromechanical properties.

**Table 33:** Micromechanical properties of AAM 60B40S 10 mol/L (sample T7), surface T7_01.

Micromechanical properties	
Hardness (Hv)	57,72
Modulus of elasticity (GPa)	15,11

**Fig. 39:**
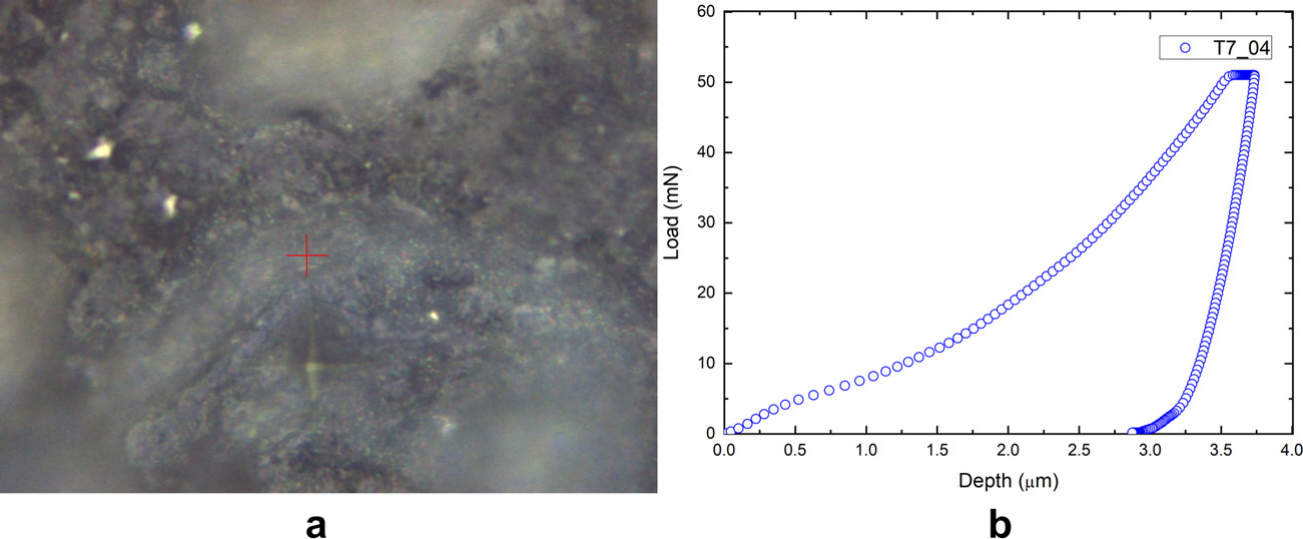
Nanoindentation test results of AAM 60B40S 10 mol/L (sample T7), surface T7_04 (a) OM image and (b) P-h curve.

The Fig. 39 shows the nanoindentation test results of AAM 60B40S 10 mol/L (sample T7), for surface T7_04. The Fig. 39(a) shows OM image and Fig. 39(b) shows P-h curve. The Table 34 shows micromechanical properties.

**Table 34:** Micromechanical properties of AAM 60B40S 10 mol/L (sample T7), surface T7_04.

Micromechanical properties	
Hardness (Hv)	20,07
Modulus of elasticity (GPa)	7,22

**Fig. 40:**
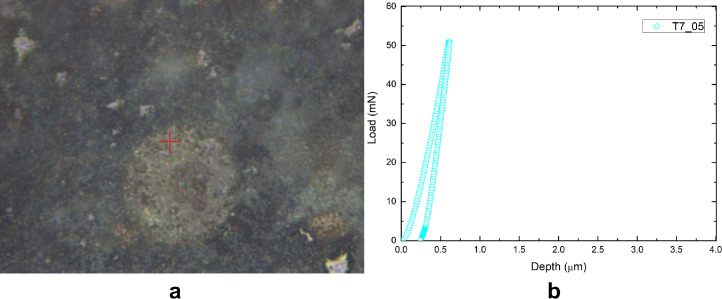
Nanoindentation test results of AAM 60B40S 10 mol/L (sample T7), surface T7_05 (a) OM image and (b) P-h curve.

The Fig. 40 shows the nanoindentation test results of AAM 60B40S 10 mol/L (sample T7), for surface T7_05. The Fig. 40(a) shows OM image and Fig. 40(b) shows P-h curve. The Table 35 shows micromechanical properties.

**Table 35:** Micromechanical properties of AAM 60B40S 10 mol/L (sample T7), surface T7_05.

Micromechanical properties	
Hardness (Hv)	589,62
Modulus of elasticity (GPa)	85,61

#### 2.1.6 AAM 60B40S 15 mol/L (sample T10)

**Fig. 41:**
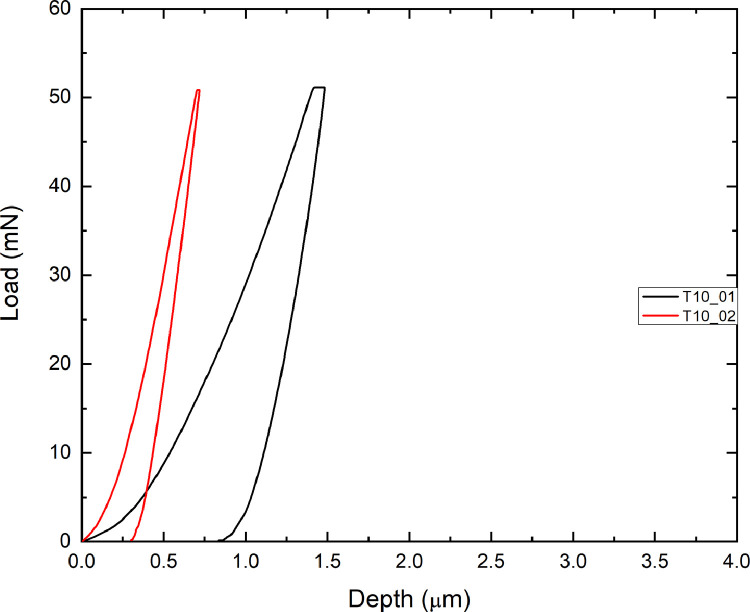
P-h curves for sample T10.

The Fig. 41 shows the load-depth (P-h) curves for sample T10 (60B40S 15 mol/L), grouped in a single graph.

**Fig. 42:**
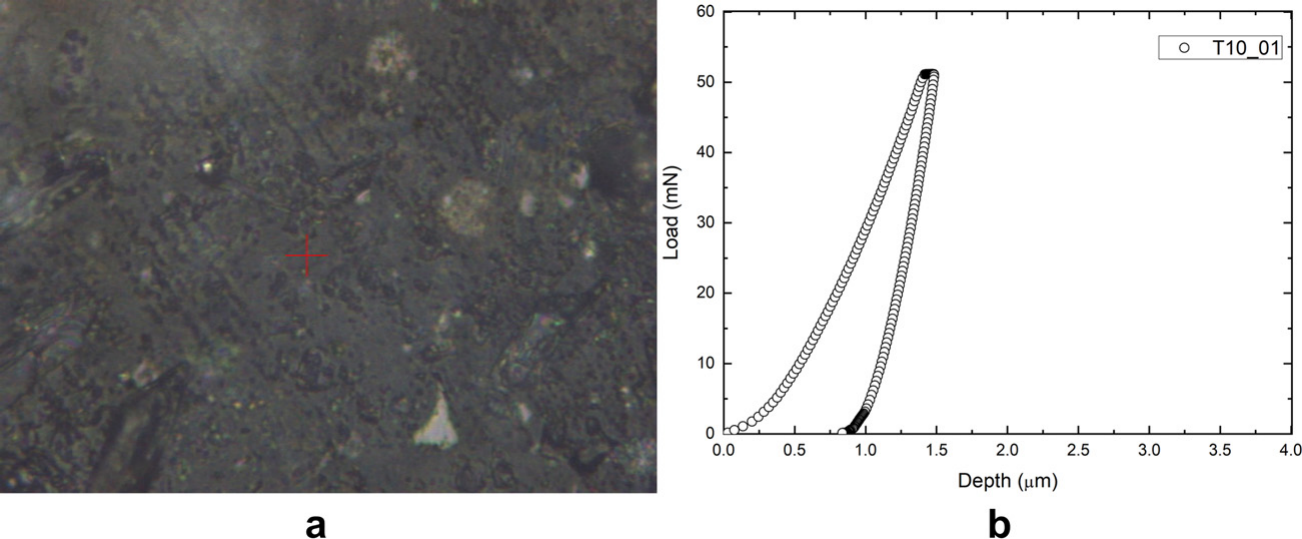
Nanoindentation test results of AAM 60B40S 15 mol/L (sample T10), surface T10_01 (a) OM image and (b) P-h curve.

The Fig. 42 shows the nanoindentation test results of AAM 60B40S 15 mol/L (sample T10), for surface T10_01. The Fig. 42(a) shows OM image and Fig. 42(b) shows P-h curve. The Table 36 shows micromechanical properties.

**Table 36:** Micromechanical properties of AAM 60B40S 15 mol/L (sample T10), surface T10_01.

Micromechanical properties	
Hardness (Hv)	89,36
Modulus of elasticity (GPa)	20,83

**Fig. 43:**
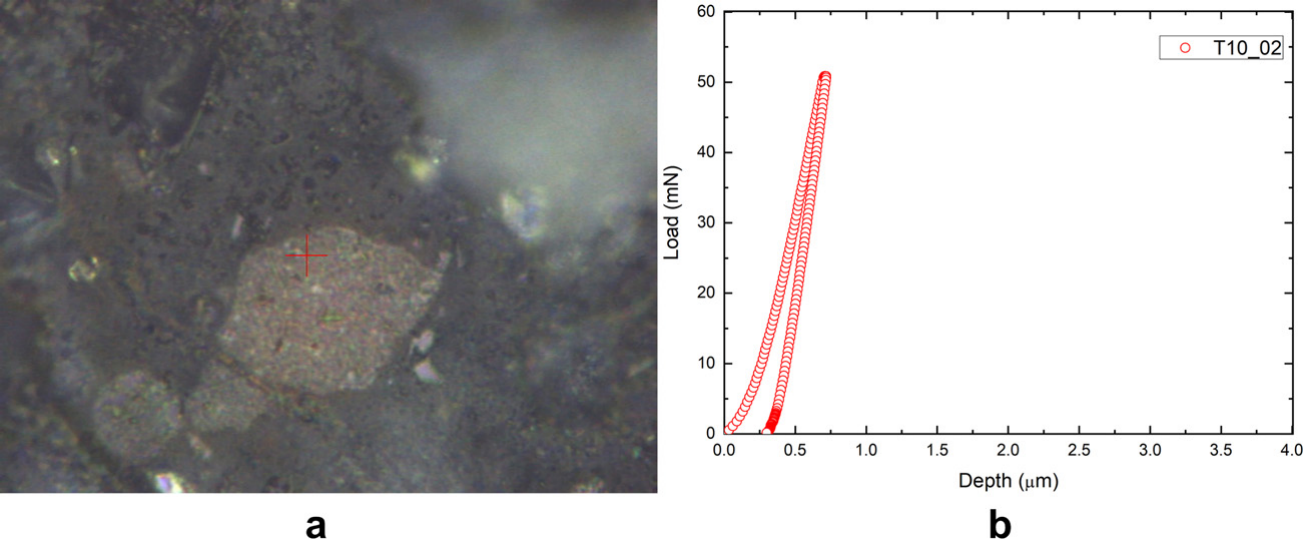
Nanoindentation test results of AAM 60B40S 15 mol/L (sample T10), surface T10_02 (a) OM image and (b) P-h curve.

The Fig. 43 shows the nanoindentation test results of AAM 60B40S 15 mol/L (sample T10), for surface T10_02. The Fig. 43(a) shows OM image and Fig. 43(b) shows P-h curve. The Table 37 shows micromechanical properties.

**Table 37:** Micromechanical properties of AAM 60B40S 15 mol/L (sample T10), surface T10_02.

Micromechanical properties	
Hardness (Hv)	371,51
Modulus of elasticity (GPa)	61,89

### 2.2 Microstructural analysis

#### 2.2.1 Microstructure by SEM

Fig. 44, Fig. 45, Fig. 46, Fig. 47, Fig. 48, Fig. 49, Fig. 50, Fig. 51, Fig. 52, Fig. 53, Fig. 54, Fig. 55 show SEM images of T1 to T12 samples at x1000 and x3000 magnifications.

**Fig. 44:**
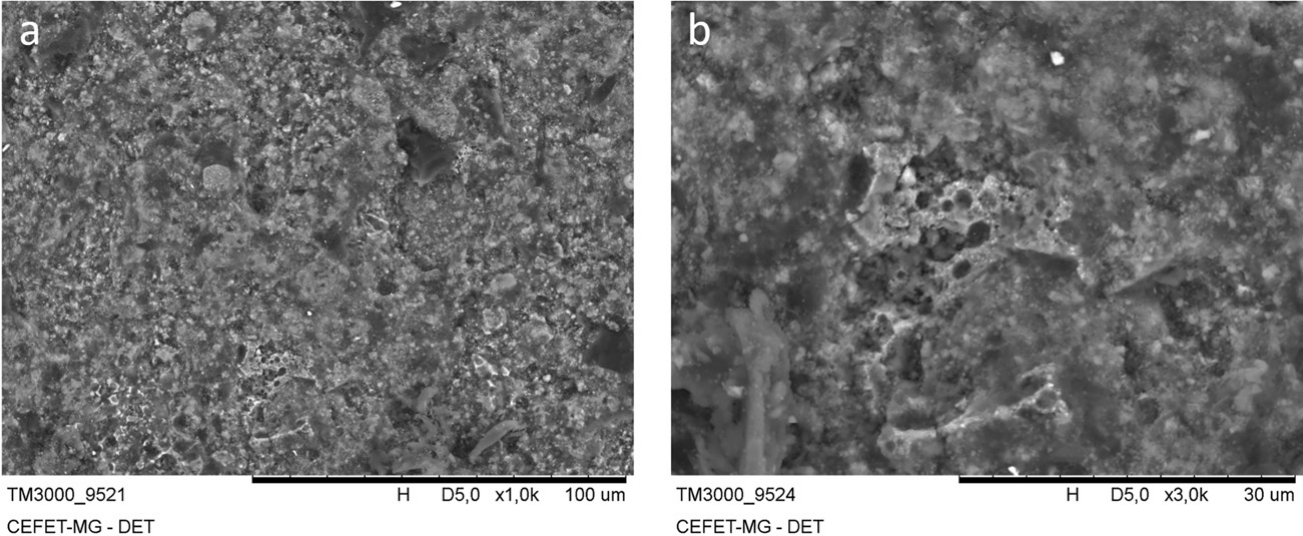
SEM images of AAM 60B40S 0 mol/L (T1 sample) at (a) x1000 and (b) x3000 magnifications.

**Fig. 45:**
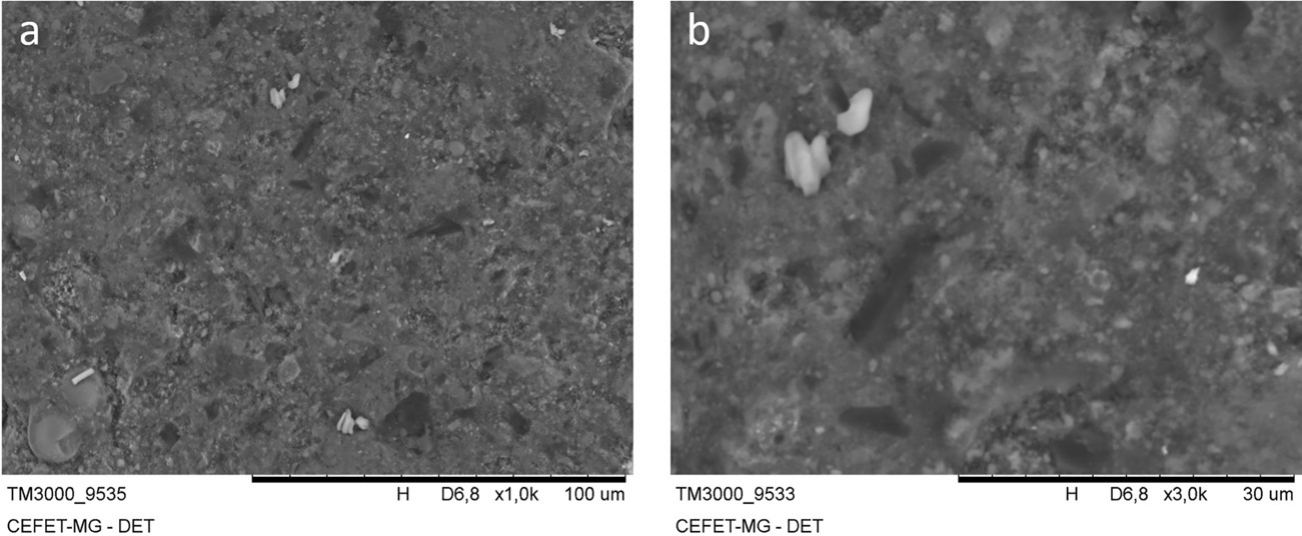
SEM images of AAM 50B50S 0 mol/L (T2 sample) at (a) x1000 and (b) x3000 magnifications.

**Fig. 46:**
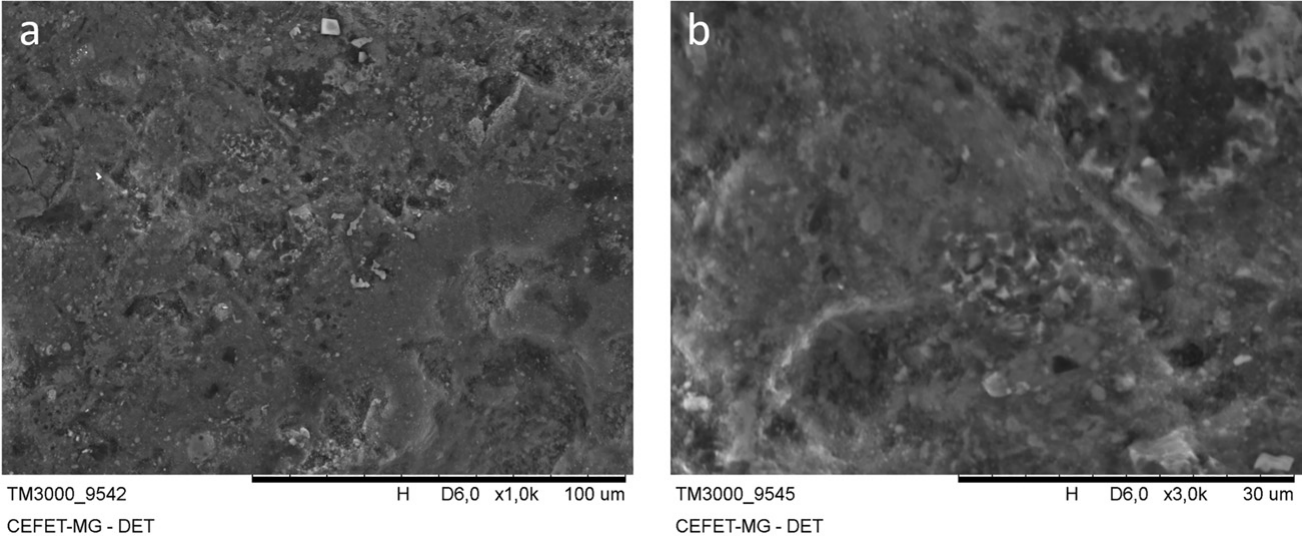
SEM images of AAM 40B60S 0 mol/L (T3 sample) at (a) x1000 and (b) x3000 magnifications.

**Fig. 47:**
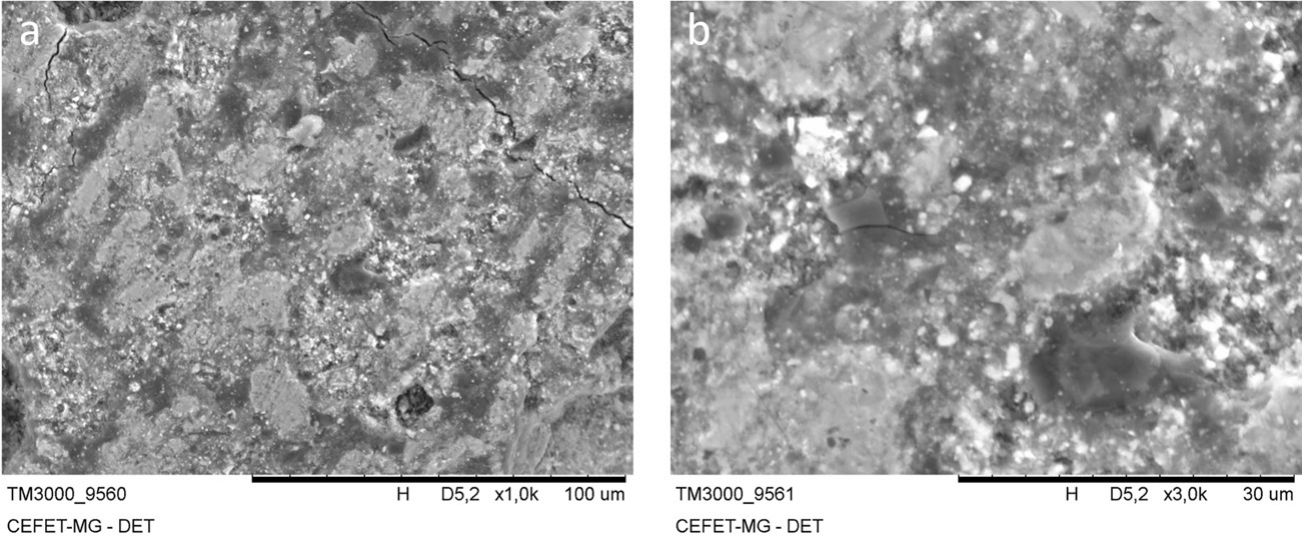
SEM images of AAM 60B40S 5 mol/L (T4 sample) at (a) x1000 and (b) x3000 magnifications.

**Fig. 48:**
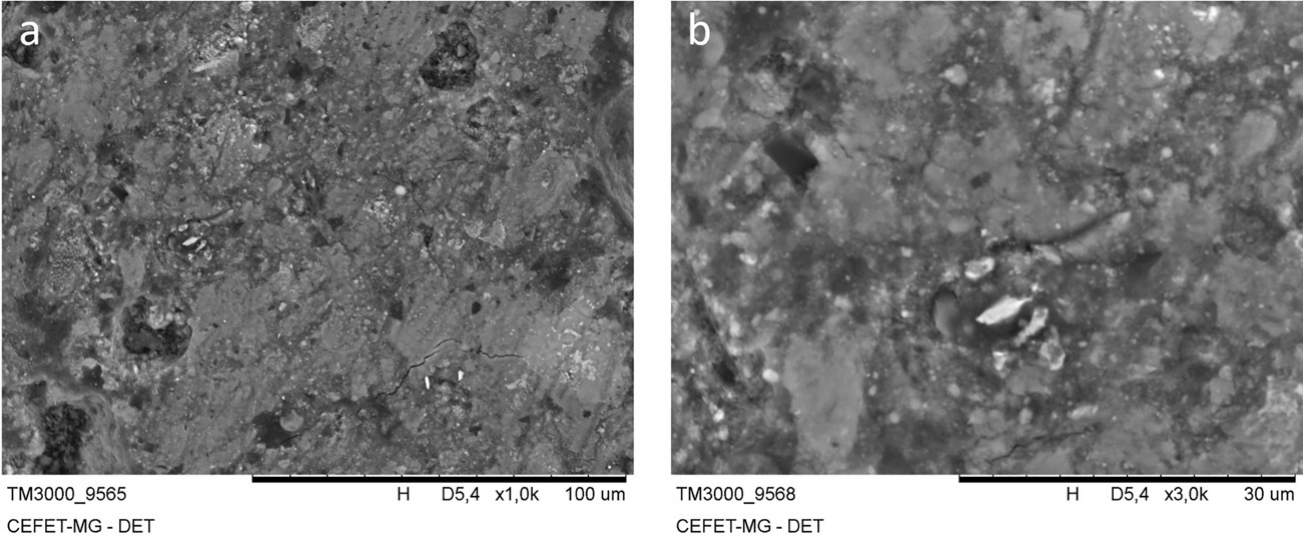
SEM images of AAM 50B50S 5 mol/L (T5 sample) at (a) x1000 and (b) x3000 magnifications.

**Fig. 49:**
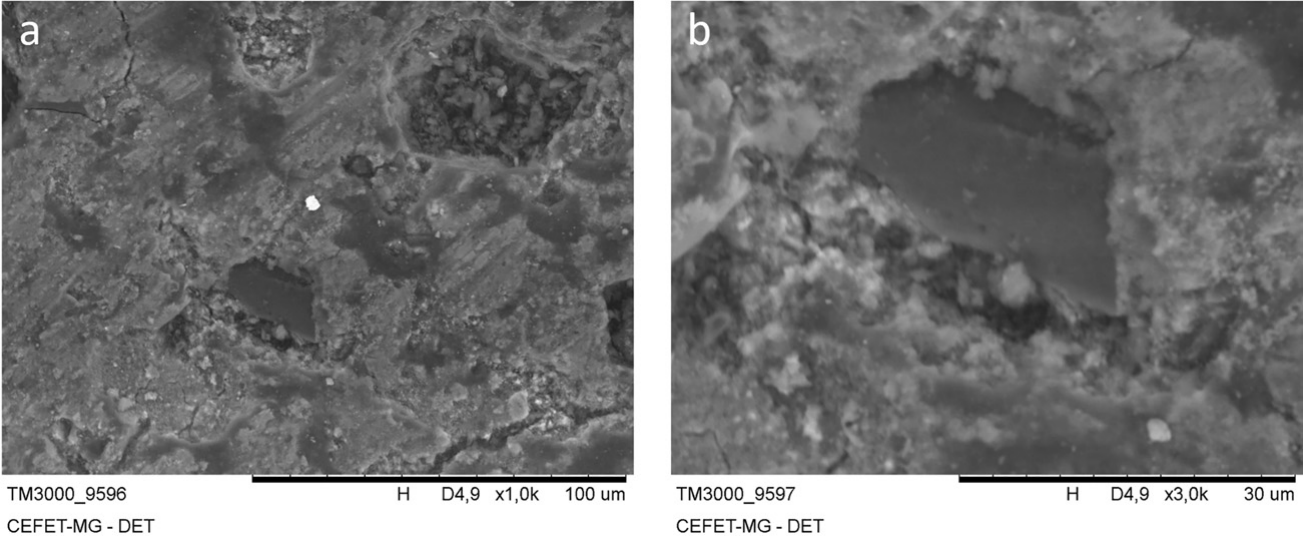
SEM images of AAM 40B60S 5 mol/L (T6 sample) at (a) x1000 and (b) x3000 magnifications.

**Fig. 50:**
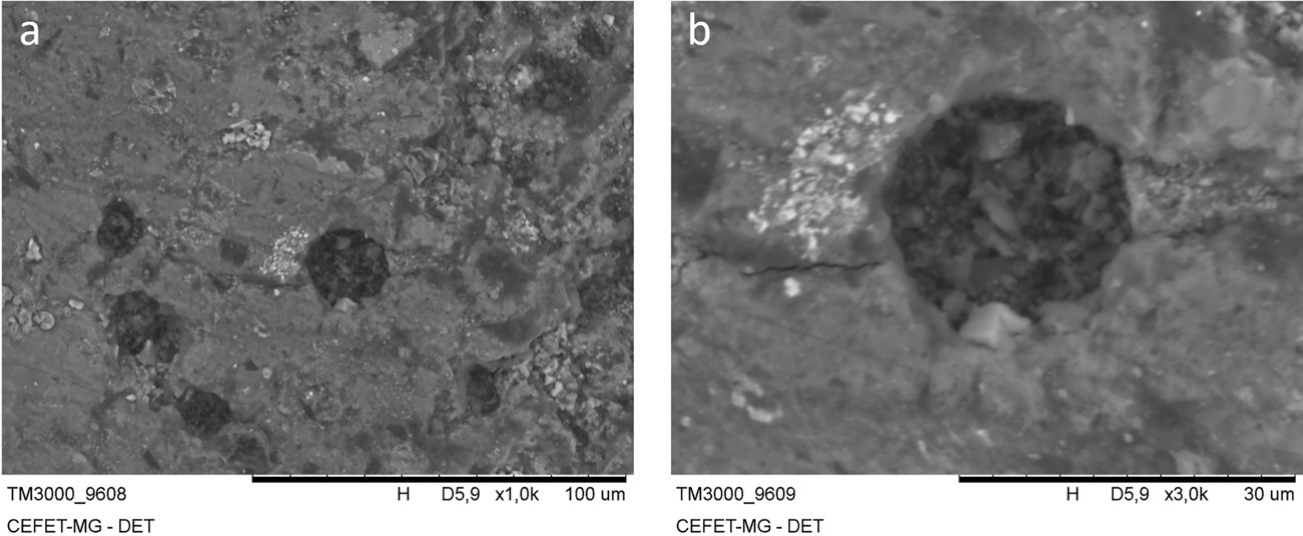
SEM images of AAM 60B40S 10 mol/L (T7 sample) at (a) x1000 and (b) x3000 magnifications.

**Fig. 51:**
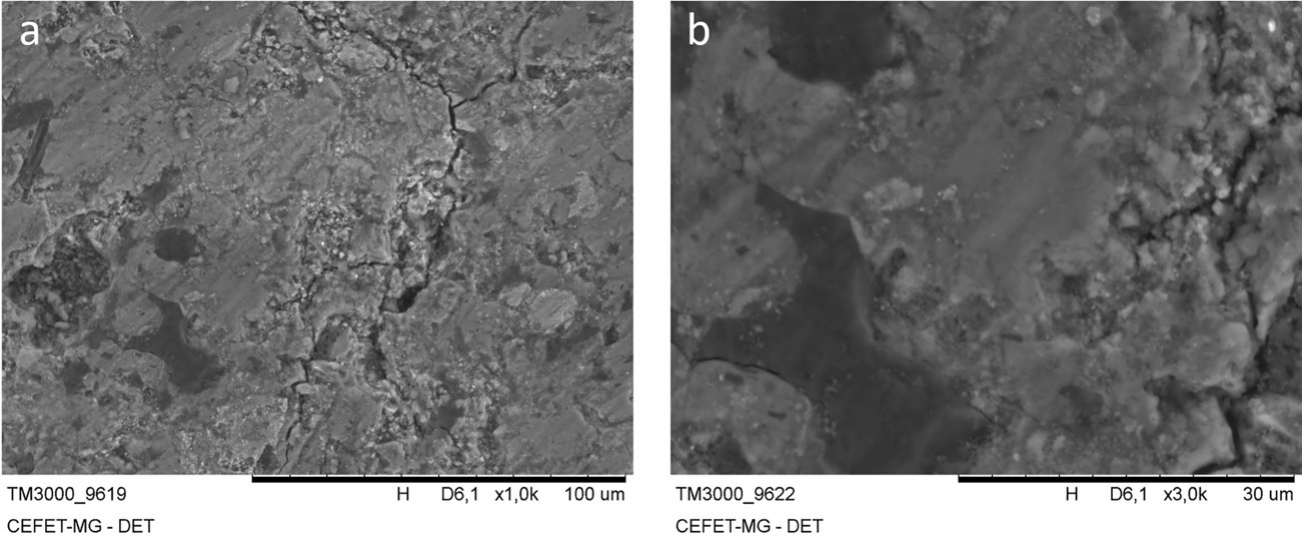
SEM images of AAM 50B50S 10 mol/L (T8 sample) at (a) x1000 and (b) x3000 magnifications.

**Fig. 52:**
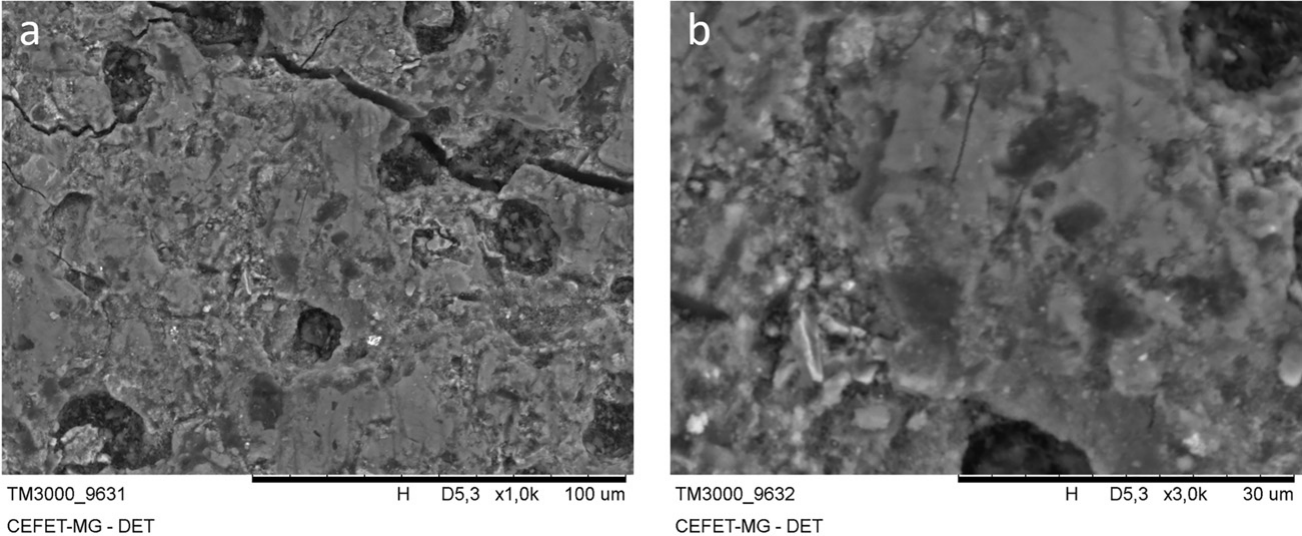
SEM images of AAM 40B60S 10 mol/L (T9 sample) at (a) x1000 and (b) x3000 magnifications.

**Fig. 53:**
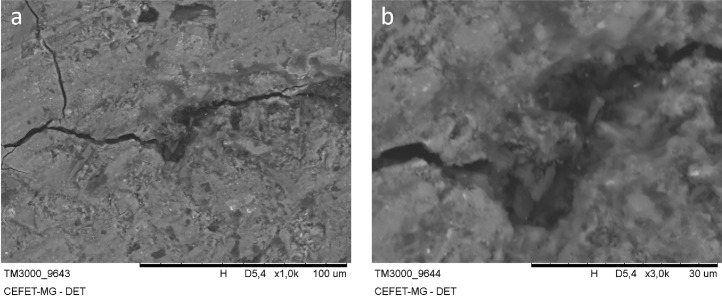
SEM images of AAM 60B40S 15 mol/L (T10 sample) at (a) x1000 and (b) x3000 magnifications.

**Fig. 54:**
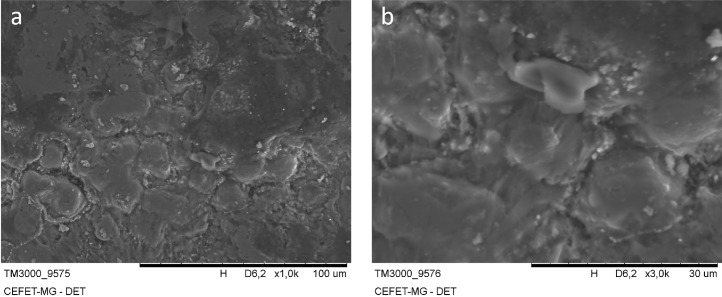
SEM images of AAM 50B50S 15 mol/L (T11 sample) at (a) x1000 and (b) x3000 magnifications.

**Fig. 55:**
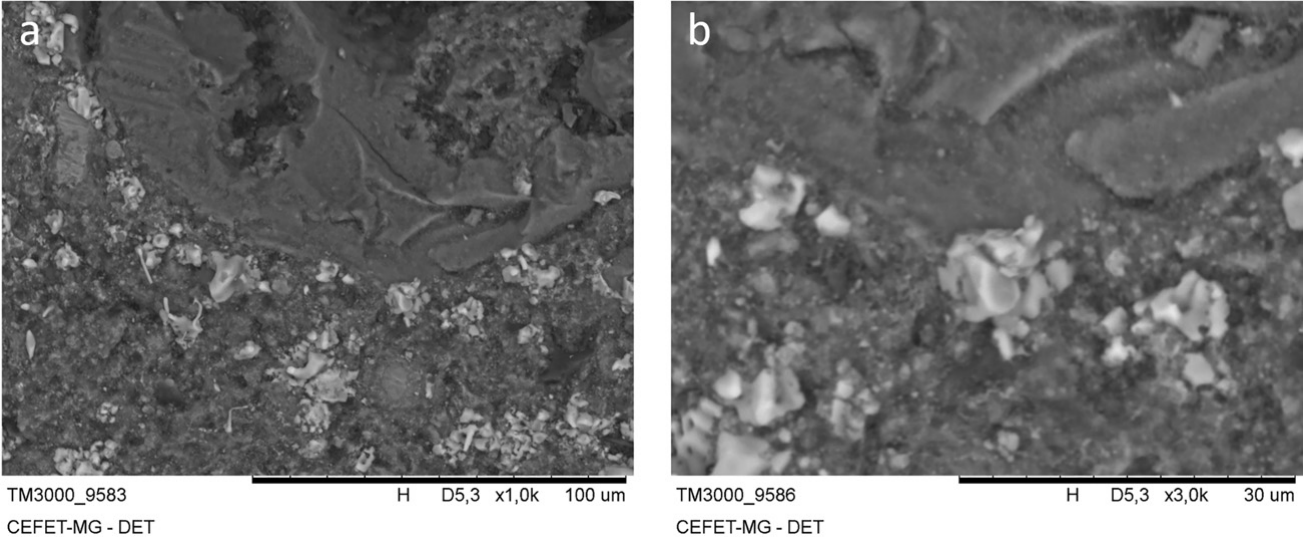
SEM images of AAM 40B60S 15 mol/L (T12 sample) at (a) x1000 and (b) x3000 magnifications.

#### 2.2.2 Microstructure by OM

Fig. 56, Fig. 57, Fig. 58, Fig. 59, Fig. 60, Fig. 61, Fig. 62, Fig. 63, Fig. 64, Fig. 65, Fig. 66, Fig. 67 show OM images with polarized light of T1 to T12 samples.

**Fig. 56:**
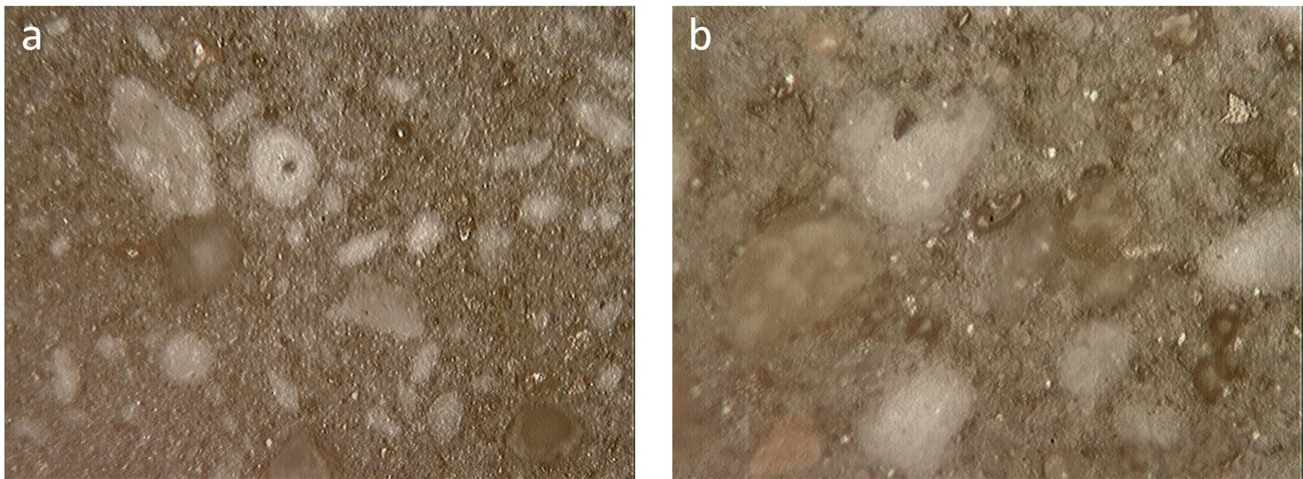
OM images of AAM 60B40S 0 mol/L (T1 sample) at (a) x100 and (b) x400 magnifications.

**Fig. 57:**
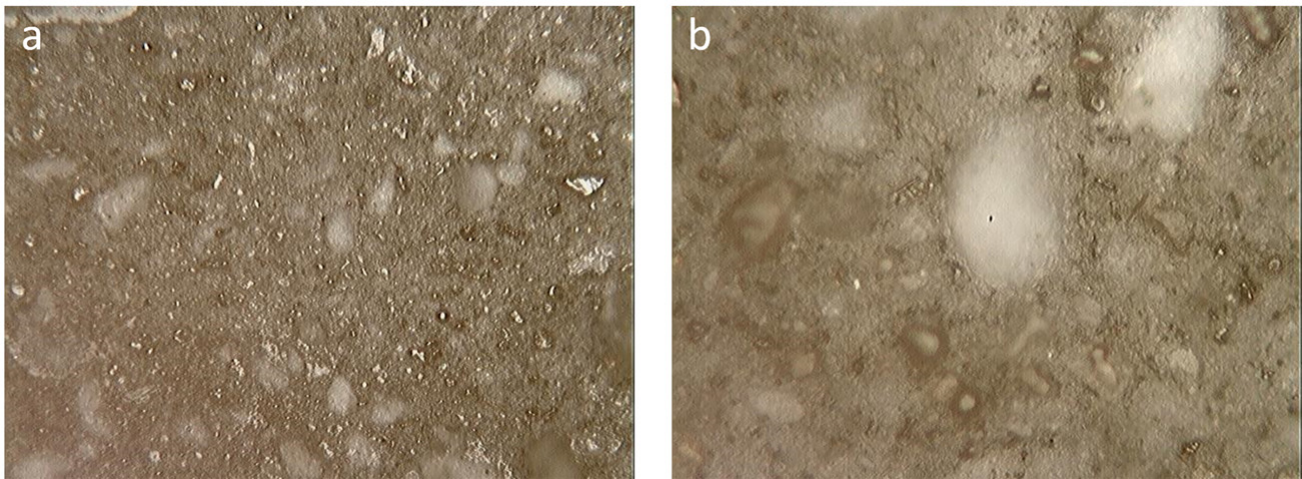
OM images of AAM 50B50S 0 mol/L (T2 sample) at (a) x100 and (b) x400 magnifications.

**Fig. 58:**
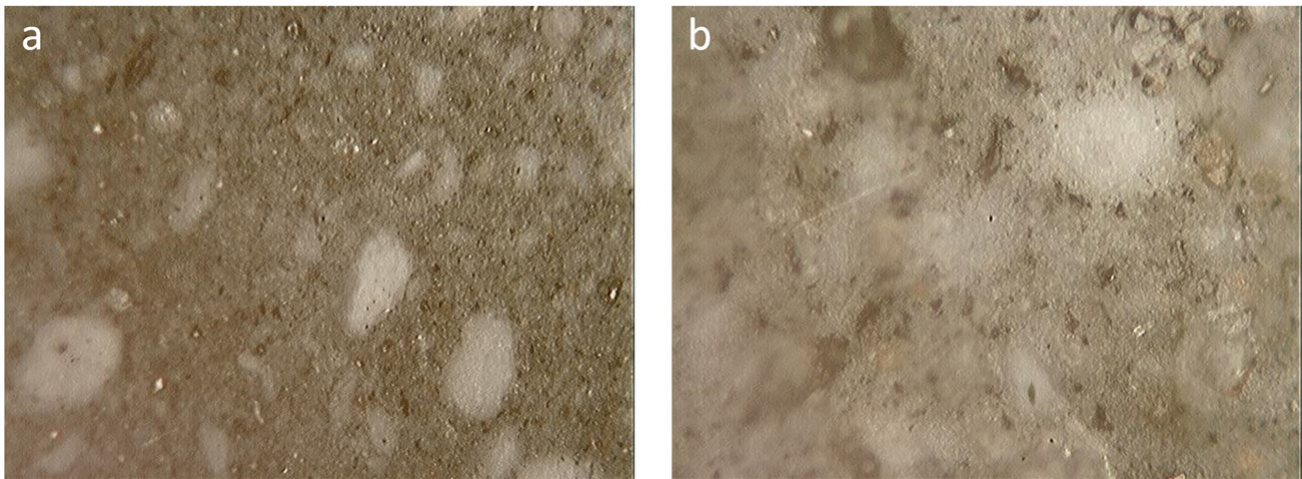
OM images of AAM 40B60S 0 mol/L (T3 sample) at (a) x100 and (b) x400 magnifications.

**Fig. 59:**
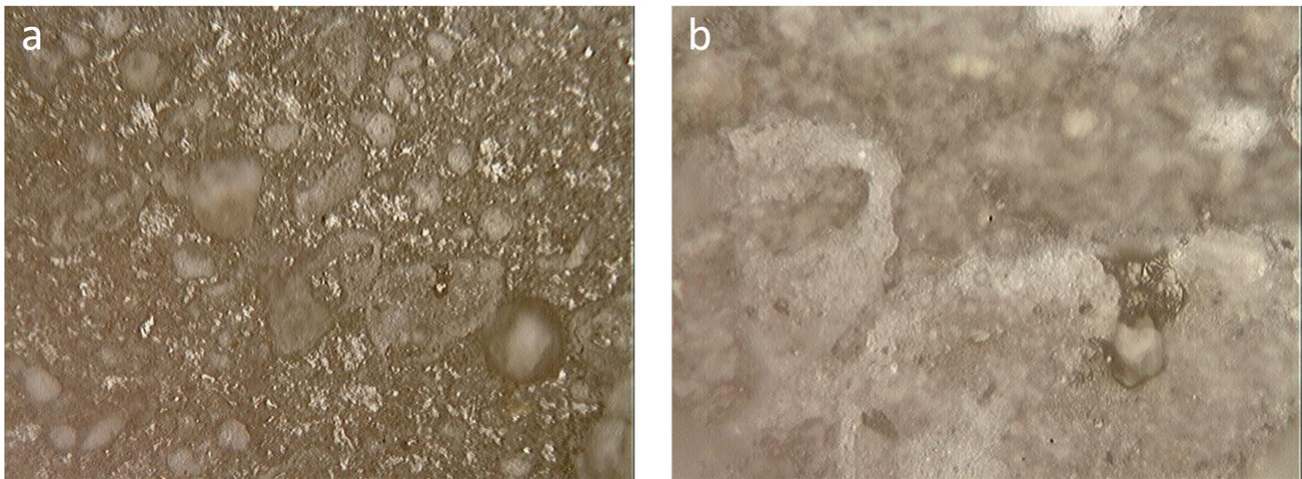
OM images of AAM 60B40S 5 mol/L (T4 sample) at (a) x100 and (b) x400 magnifications.

**Fig. 60:**
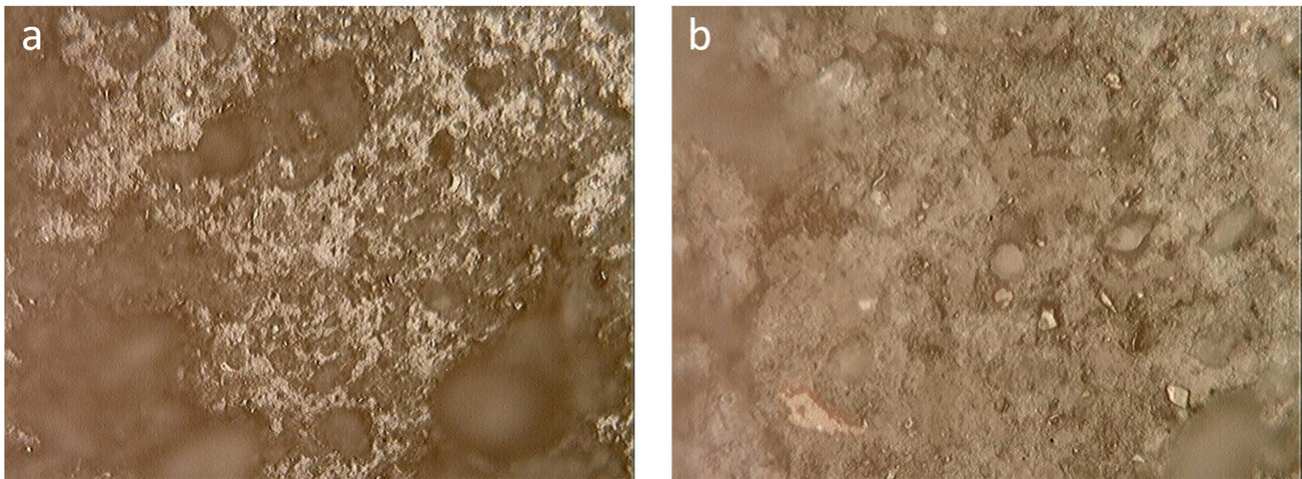
OM images of AAM 50B50S 5 mol/L (T5 sample) at (a) x100 and (b) x400 magnifications.

**Fig. 61:**
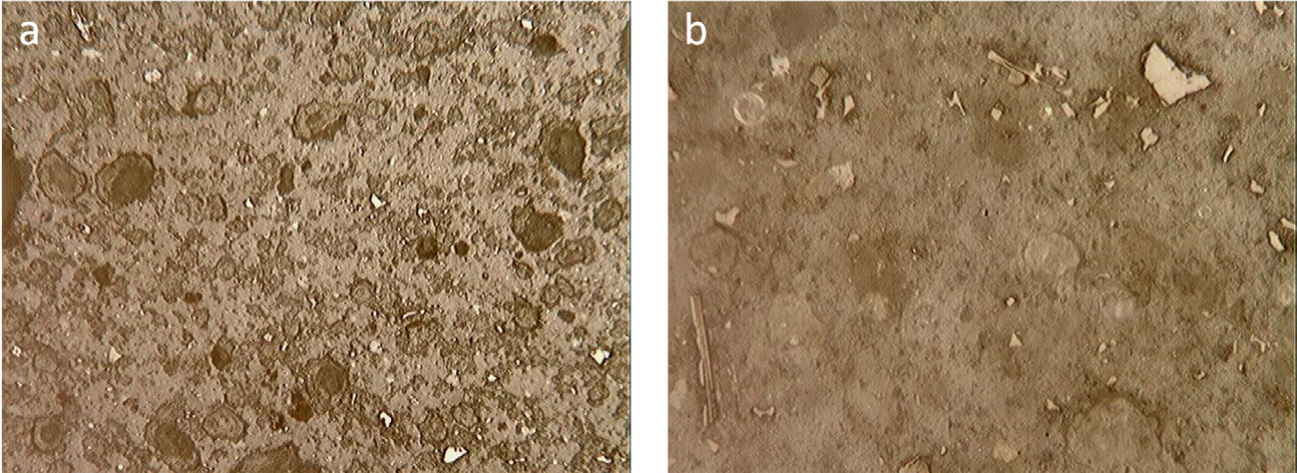
OM images of AAM 40B60S 5 mol/L (T6 sample) at (a) x100 and (b) x400 magnifications.

**Fig. 62:**
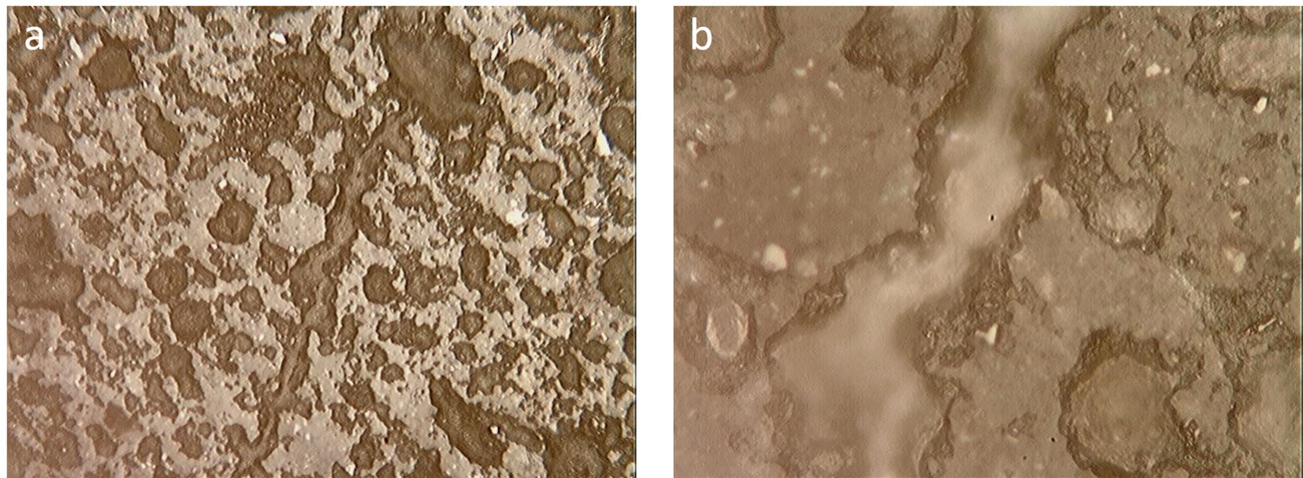
OM images of AAM 60B40S 10 mol/L (T7 sample) at (a) x100 and (b) x400 magnifications.

**Fig. 63:**
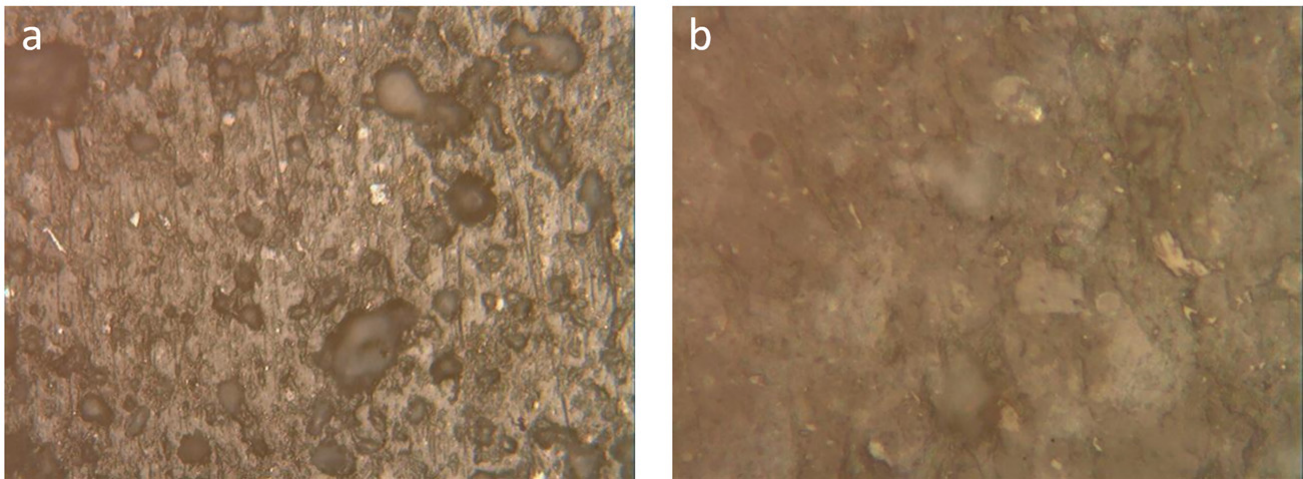
OM images of AAM 50B50S 10 mol/L (T8 sample) at (a) x100 and (b) x400 magnifications.

**Fig. 64:**
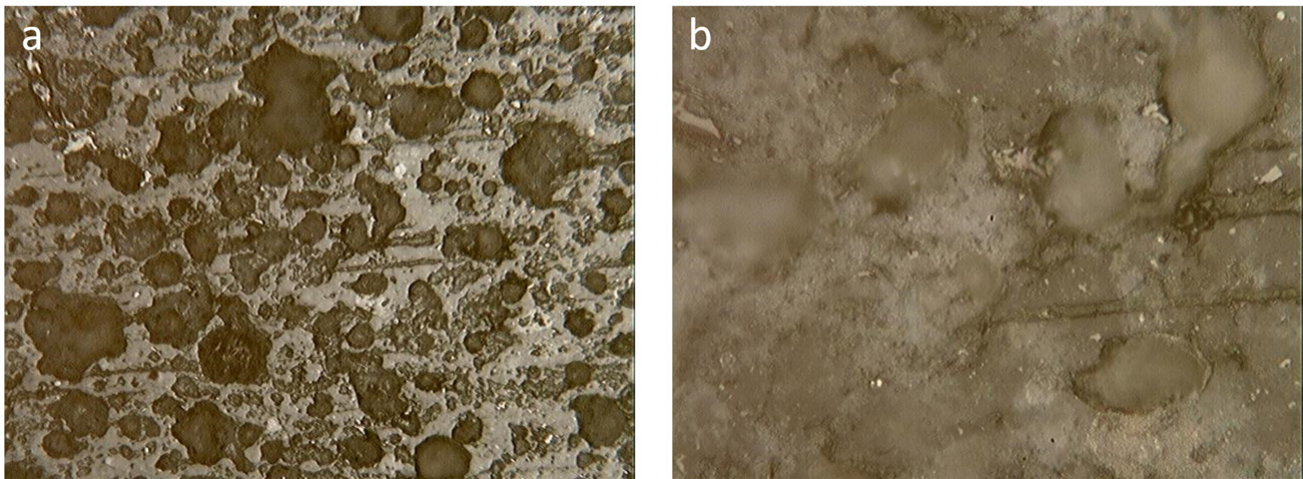
OM images of AAM 40B60S 10 mol/L (T9 sample) at (a) x100 and (b) x400 magnifications.

**Fig. 65:**
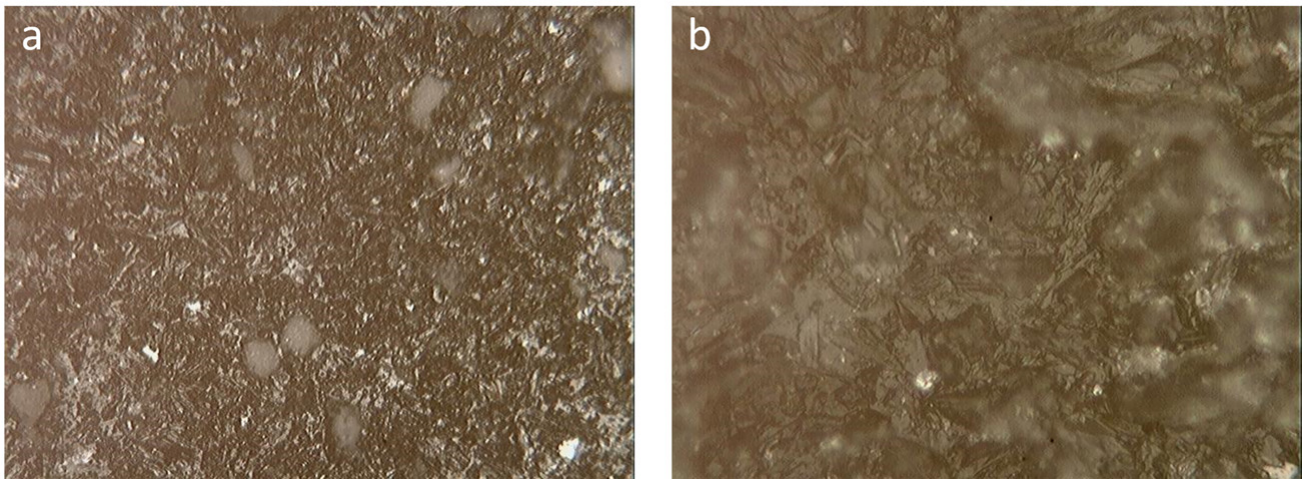
OM images of AAM 60B40S 15 mol/L (T10 sample) at (a) x100 and (b) x400 magnifications.

**Fig. 66:**
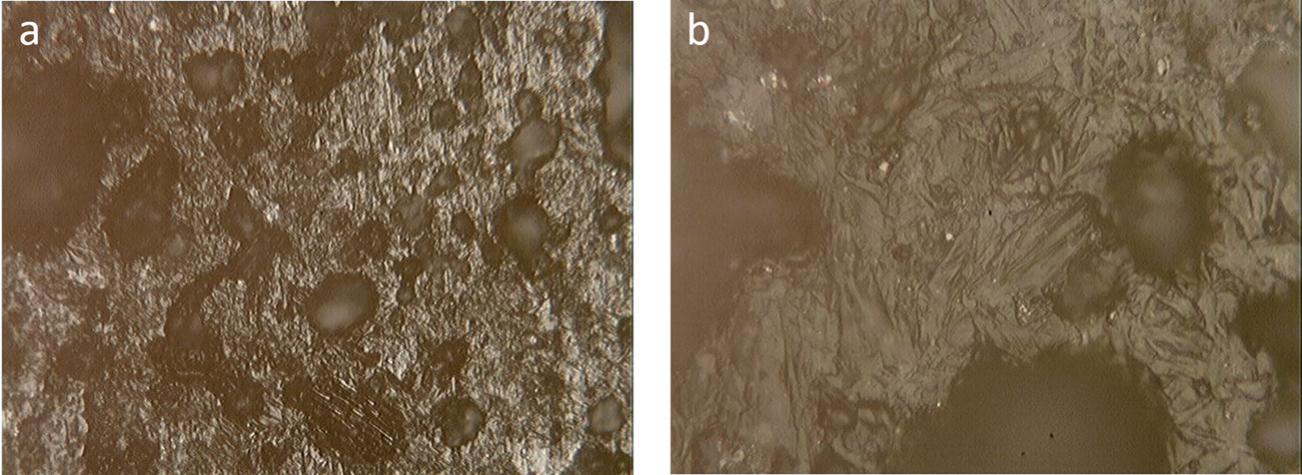
OM images of AAM 50B50S 15 mol/L (T11 sample) at (a) x100 and (b) x400 magnifications.

**Fig. 67:**
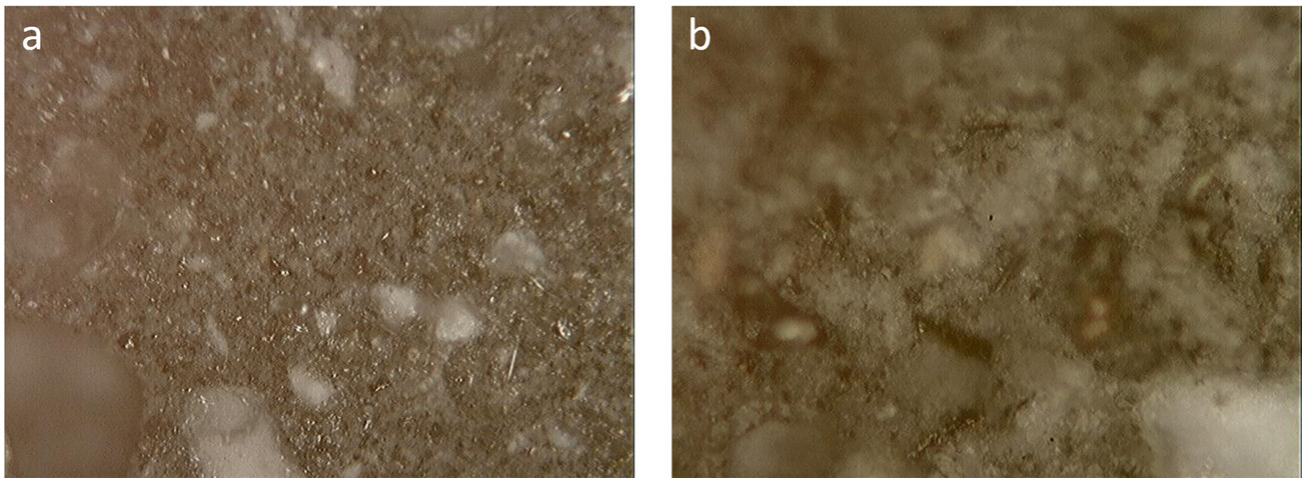
OM images of AAM 40B60S 15 mol/L (T12 sample) at (a) x100 and (b) x400 magnifications.

### 2.3 Mix proportion

Table 38 shows the proportions of constituents, which are biomass ash, silica fume and NaOH solution. The ratio of precursors (B and S) were defined based on the total fraction equal to 1. The identification of the specimens was established based on the proportions.

**Table 38:** Raw materials proportions for the AAMs production.

Sample	B ratio	S ratio	NaOH solution (mol/L)
AAM 60B40S 0 mol/L (T1)	0.6	0.4	0
AAM 50B50S 0 mol/L (T2)	0.5	0.5	0
AAM 40B60S 0 mol/L (T3)	0.4	0.6	0
AAM 60B40S 5 mol/L (T4)	0.6	0.4	5
AAM 50B50S 5 mol/L (T5)	0.5	0.5	5
AAM 40B60S 5 mol/L (T6)	0.4	0.6	5
AAM 60B40S 10 mol/L (T7)	0.6	0.4	10
AAM 50B50S 10 mol/L (T8)	0.5	0.5	10
AAM 40B60S 10 mol/L (T9)	0.4	0.6	10
AAM 60B40S 15 mol/L (T10)	0.6	0.4	15
AAM 50B50S 15 mol/L (T11)	0.5	0.5	15
AAM 40B60S 15 mol/L (T12)	0.4	0.6	15

## 3 Experimental Design, Materials and Methods

### 3.1 Materials and proportions

The AAM were originally produced by Lara [4] using eucalyptus ash (B), silica fume (S), distilled water and sodium hydroxide solution. The NaOH solution, B and S in the respective proportions were carefully mixed and casted in molds. After hardening, the samples were cured in laboratory conditions until the advanced age of 1000 days, when they were tested.

### 3.2 Methodology

#### 3.2.1 Sample preparation and microstructure analysis

From each of the twelve proportions described above, a sample with 30mm in diameter and 4mm in thickness was produced, which was subjected to cleaning using isopropyl alcohol to remove the remaining moisture. Twelve samples were prepared, divided into equal parts in the form of semicircles and one of the halves was embedded in acrylic resin. In the end, twelve embedded samples were obtained, which were subjected to sanding and polishing, in order to regularize the surface and remove imperfections. The embedded samples destined for the scanning electron microscopy (SEM) test were subjected to sanding with 120#, 240#, 320#, 400# and 600# sandpaper and subsequently polished using a 9 μm diamond paste. The samples used in the optical microscopy and nanoindentation tests were subjected to a new sanding step, with 600# and 1,000# grit sandpaper and polished again with the same diamond paste. The microstructure was evaluated using low vacuum SEM, Hitachi, model TM 3000, backscattered electron detection, with magnification from 15 to x30,000, acceleration of 5 and 15kV, and Kontrol OM with polarized light and MCDE-5 A digital camera coupled.

#### 3.2.2 Micromechanical characterization

The micromechanical characterization was based on the determination of the hardness and modulus of elasticity of the phases present in the AAM microstructure, using a Shimadzu ultra-microdurameter, model DUH-211S, with instrumented penetration, Vickers pyramidal indenter, and optical system with lens of the microscope (x500), objective (x50) and ocular (x10). The equipment makes it possible to measure the load (P) applied as a function of the depth of penetration (h) dynamically, resulting in curves of the P-h type. This method allows obtaining information about the mechanical properties in microregions and loads of the order of 0.10 mN. Several measurements were performed for each sample. Six AAM samples, out of a total of twelve produced, were analyzed. Samples T1, T4, T5, T6, T7 and T10 were strategically chosen because they represent AAM without alkaline activation (T1), AAM with low and medium alkaline activation (T4, T5, T6 and T7) and AAM with high alkaline activation (T10).

## CRediT authorship contribution statement

**Paulo Roberto Ribeiro Soares Junior:** Data curation, Writing – original draft, Visualization. **Ederson Jose da Silva:** Methodology, Investigation, Validation. **Augusto Cesar da Silva Bezerra:** Conceptualization, Supervision, Project administration, Resources, Funding acquisition.

## Declaration of Competing Interest

The authors declare that they have no known competing financial interests or personal relationships that could have appeared to influence the work reported in this paper.

## Data Availability

Raw and analyzed data on the micromechanical behavior and microstructure of 1000-day-old alkali-activated materials (Original data) (Mendeley Data).
